# Moving targets in drug discovery

**DOI:** 10.1038/s41598-020-77033-x

**Published:** 2020-11-19

**Authors:** Barbara Zdrazil, Lars Richter, Nathan Brown, Rajarshi Guha

**Affiliations:** 1 Division of Drug Design and Medicinal Chemistry, Department of Pharmaceutical Chemistry, University of Vienna, Althanstrasse 14, 1090 Vienna, Austria; 2 BenevolentAI, 4-8 Maple Street, London, W1T 5HD UK; 3 Vertex Pharmaceuticals, 50 Northern Avenue, Boston, MA 02210 USA

**Keywords:** Computational biology and bioinformatics, Drug discovery, Diseases

## Abstract

Drug Discovery is a lengthy and costly process and has faced a period of declining productivity within the last two decades resulting in increasing importance of integrative data-driven approaches. In this paper, data mining and integration is leveraged to inspect target innovation trends in drug discovery. The study highlights protein families and classes that have received more attention and those that have just emerged in the scientific literature, thus highlighting novel opportunities for drug intervention. In order to delineate the evolution of target-driven research interest from a biological perspective, trends in biological process annotations from Gene Ontology and disease annotations from DisGeNET are captured. The analysis reveals an increasing interest in targets related to immune system processes, and a recurrent trend for targets involved in circulatory system processes. At the level of diseases, targets associated with cancer-related pathologies, intellectual disability, and schizophrenia are increasingly investigated in recent years. The methodology enables researchers to capture trends in research attention in target space at an early stage during the drug discovery process. Workflows, scripts, and data used in this study are publicly available from https://github.com/BZdrazil/Moving_Targets. An interactive web application allows the customized exploration of target, biological process, and disease trends (available at https://rguha.shinyapps.io/MovingTargets/).

## Introduction

The organization of proteins (and genes) into families, is usually achieved through phylogeny (amino acid sequence similarity) and—to a lesser extent—by similarity of secondary structures. The assumption is that proteins belonging to the same family share a common function which will also be encoded in the protein sequences and their folds.

Apart from the general desire to organize information, such indexing and grouping of proteins is especially relevant in drug discovery. If we understand how protein modulation, e.g. by small molecules works for a particular protein, we also move closer towards understanding the effects of modulating a whole group of related proteins. Thus, structure–function relationships are often carried out at the protein family level^1,2^. Somewhat contradictory to this observation is the phenomenon of polypharmacology, where a molecule interacts with multiple (often unrelated) targets (either wanted or unwanted)^3–5^. Therefore, across target families, close similarities in binding patterns appear to occur for similar ligands. This has been explored systematically for the identification of novel ligand-target associations^6–8^. A straightforward application of exploring polypharmacology of drugs is for repurposing reasons^9^, especially in the area of rare diseases^10^.

Setting aside the goal of unraveling ligand–protein interactions, another emerging application of target (family) indexing and characterization in drug discovery is the desire to capture the past, current, and (potential) future degree of research interest in main drug target families. Until now, exploratory studies of research attention across target families have been carried out mainly from a static point of view with only a few analyses including the time dimension. In an analysis article from 2011 by Rask-Andersen and colleagues^11^, the authors looked into trends in the exploitation of new drug targets over a period of 30 years and have identified three innovation peaks. Interestingly, when exploring drug-target networks their analysis revealed that newer molecular entities tend to form isolated networks pointing towards a tendency to interact with new drug targets and potentially possessing new modalities for treatment.

In a more recent analysis by Santos et al.^12^ the authors highlight the main drug efficacy target families, which together account for approximately 44% of all human drug targets: G-protein coupled receptors (GPCRs), kinases, ion channels, and nuclear receptors. The authors also present a temporal view on innovation patterns in these privileged protein classes by analyzing the proportion of approved drugs for each target class comparing different time frames. In addition, patterns of innovation in different therapeutic areas are represented in a timewise manner, but independent of the target class.

Since 2014, a strategic effort by the US National Institutes of Health termed Illuminating the Druggable Genome (IDG) initiative has focused on exploring currently understudied, but potentially druggable, proteins^13^. In order to be able to define the degree to which a target is comparatively well studied or understudied, the existing knowledge on current associations between compounds (or drugs), targets, Mechanism of Action (MoA), and disease were systematically collected and mapped by this initiative. These efforts resulted in defining so-called Target Developmental Level (TDL) categories by which targets can be classified according to the depth of investigations undertaken for a particular target (by uniting available chemical, biological and clinical information). The four categories in the TDL scheme, T_clin_ (clinic: drug annotation(s) with approved MoA), T_chem_ (chemistry: binding small molecules with high potency), T_bio_ (biology: disease annotation(s) available), and T_dark_ (dark genome: not falling into one of the other categories), are thereafter representing different (decreasing) stages of target innovation. A multimodal web interface called PHAROS enables intuitive access to the IDG knowledgebase^14^. A similar European initiative, called Open Targets, uses human genetics and genomics data for systematic drug target identification and prioritization^15^.

The immense pool of small compound data spans two main data repertoires where preclinical data is frequently reported: scientific literature and patents. A thorough analysis of trends in publishing behavior of bioactivity compounds for novel targets (rather literature or patents first) was delivered by Ashenden et al.^16^. Preclinical data can guide decision making at earlier stages in the drug discovery pipeline since it reflects trends in research attention within the community. It might help researchers to base their research endeavors on evidence-based criteria and direct their interest towards understudied proteins or core scaffolds^17^.

In this study, major target classes are monitored over time to capture the different aspects of target innovation in drug discovery. First, target families of emerging interest in drug discovery are highlighted as reflected by an increasing number of targets, bioactivity measurements, annotated drugs, and number of publications. Second, trends in GO (Gene Ontology) biological process (BP) annotations enable us to identify biological processes of (potential) emerging attention per target family. Third, studying the evolution of disease annotations for major target families highlights popular targets of intervention for particular diseases. Finally, we undertook an attempt to test for causality between disease trends derived from targets and disease trends derived from publications (via searches of PubMed abstracts).

## Results and discussion

### Target innovation trends

We investigated protein innovation trends by inspecting different measures reflecting research attention per target family: increase or decrease of the number of unique compounds, number of drug-efficacy target annotations (playing a role in the efficacy of the drug in the indication(s) for which it is approved), number of published papers (as expressed by PMIDs), and number of unique targets. In addition, we studied the evolution of the proportions of Target Developmental Levels (TDLs) through the years for each target family.

**Figure 1:**
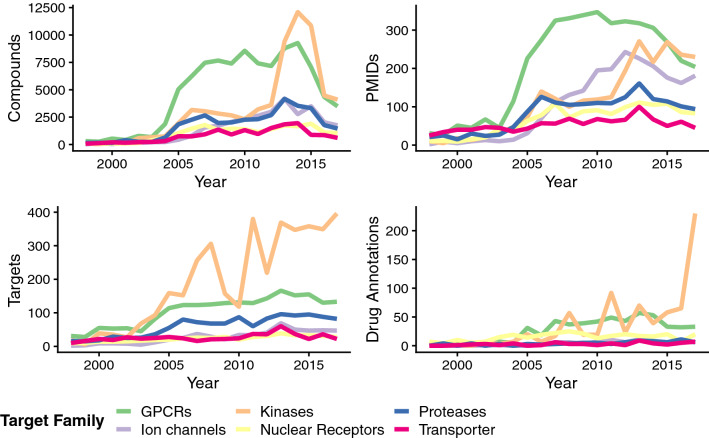
Graph showing the evolution of different measures of popularity for a respective target family over time in absolute numbers: numbers of unique compounds (“Compounds”), numbers of Pubmed IDs (“PMIDs”), numbers of targets (“Targets”), and numbers of drug-efficacy target annotations (“Drug Annotations”).

For all four measures describing protein family popularity in absolute numbers (Fig. 1), the steepest upward trends can be observed for GPCRs and kinases over 20 years (1998–2017). Although the GPCR family was the most studied in terms of unique compounds and papers from the year 2000 onwards, kinases outpaced GPCRs towards the end of the observation period (from 2013 for compound counts, and from 2015 for paper counts). Investigating the trend lines delivered by target and drug-target annotation counts (Fig. 1, bottom left and right), a less steady increase for the family of kinases can be observed since large fluctuations in these measures were taking place from 2005 onwards. Two large peaks in terms of the number of published targets can be denoted for 2011 (380 unique targets) and 2008 (306 unique targets), which is not reflected in a particularly large number of papers for these years (125 and 100 papers, respectively). Looking a bit closer at the respective numbers of targets published in the respective papers of these years, it becomes clear that these two target peaks can each be attributed to one large study, which were both dedicated to comprehensive kinase inhibitor selectivity screens^18,19^. As obvious from the steep rise in paper and compound counts from 2012/2013 onwards the two large scale screening studies (both designed by Zarrinkar et al.) might have sparked the increasing research interest in particularly this family of proteins since “the data set suggests compounds to use as tools to study kinases for which no dedicated inhibitors exist”^18^. In terms of numbers of drug-efficacy target annotations, a picture similar to that of target trends can be observed with also a peak in 2011 originating from the paper by Davis et al. (81 out of 92 total drug-target annotations in 2011). Strikingly, in 2017 a very large number of drug-target annotations (227) was reported in literature. 210 annotations out of those originate from a single publication by Klaeger et al. in which the target spectrum of 243 clinically evaluated kinase drugs was systematically analyzed^20^.

In comparison to kinases, all other target families exhibit relatively smooth increases in the different popularity measures depicted. Interestingly, GPCRs, although of emerging interest until recently, were not greatly affected by large screening studies (which is revealed by their high paper counts over the years and relatively flat compound and target trends between 2005 and 2017). Membrane proteins like GPCRs are extremely challenging targets when it comes to protein purification and crystallization. Therefore—although 33% of all small molecule drugs are targeting GPCRs—they account for only 12% of human protein drug targets^12^.

From Fig. 1 it also becomes visible that ion channels have outperformed proteases in terms of paper numbers over the years. This is not unexpected since ion channels according to Santos et al. make up the largest proportion of drug targets inspecting the individual protein families (19%)^12^. However, to date this is not so well reflected in terms of literature-reported compounds, targets and drug-efficacy target annotations (Fig. 1).

**Figure 2:**
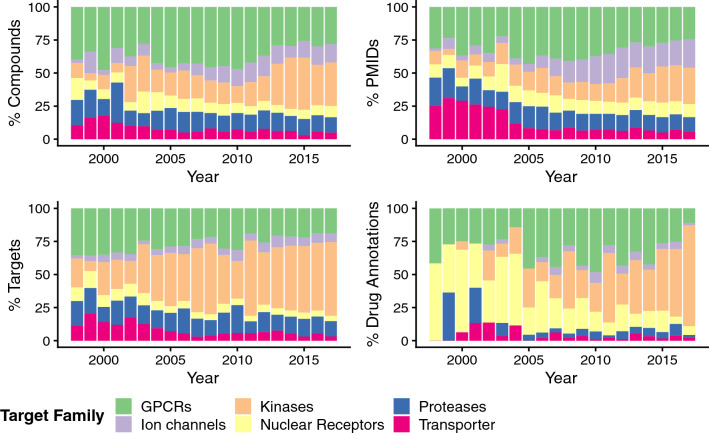
Bar charts showing the evolution of different measures of popularity for a respective target family over time expressed as proportions: percentages of compounds (“% Compounds”), percentages of Pubmed IDs (“% PMIDs”), percentages of targets (“% Targets”), and percentages of drug-efficacy target annotations (“% Drug Annotations”).

By relating the absolute numbers from target innovation trends (as represented in Fig. 1) to the respective total numbers of compounds, papers, drug-efficacy target annotations, and targets per year, relative trend measures are observed. These enable temporal comparison across the target families more easily, particularly when evaluating the early years of rational drug discovery, which tend to be relatively data poor. Notably, nuclear receptors outperformed all other target families in terms of drug-efficacy target annotations from 1998–2004, but not in terms of other popularity measures (Fig. 2). This is also reflected by inspecting the proportions of small-molecule drugs and human drug targets, discussed in Santos et al.^12^: nuclear receptor drugs make up 16% of all drugs, but only 3% of drug targets. Inspecting the proportions of drug-efficacy target annotations over the whole observation period (Fig. 2), it becomes clear that from 2005 onwards primarily GPCRs and later kinases display a relative enrichment in this popularity measure (in relation to drug-efficacy target annotations for all six target families). Notably, interest in kinases seems to be a more recent trend exemplified by the relative enrichment in all popularity measures under investigation by 2017 (Fig. 2).

**Figure 3:**
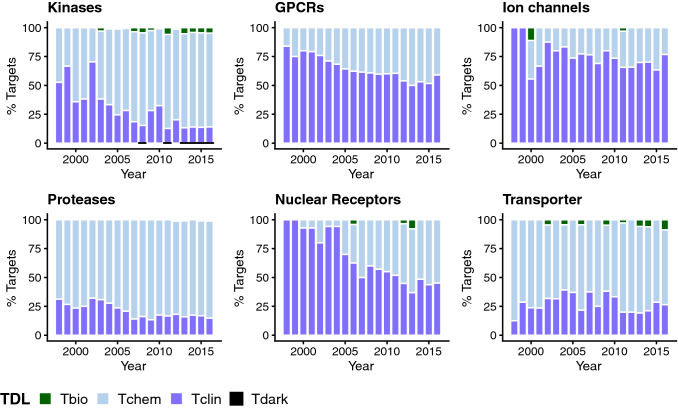
Bar charts showing the time evolution of the Target Developmental Level (“TDL”) categories across different target families.

A multi-dimensional measure of (evidence-based) research attention in particular human protein targets is provided by the Target Developmental Level (TDL) categories that were assigned by the IDG initiative^13,14^. By looking at the time evolution of TDL categories protein family-wise (Fig. 3), differences with respect to stages of drug development between the families can be captured. It has to be noted, that this visualization does not show the entirety of protein targets for each target family but reflects research endeavors, successes and future opportunities as reflected in the scientific literature (and recorded by ChEMBL). From Fig. 3 it becomes obvious that within the T_clin_ category, ion channels and GPCRs make up the largest fraction of targets, consistently over the years. The majority of well-studied ion channel and GPCR proteins are already linked to at least one approved drug by MoA and proportionally not many new targets without known drugs (categories T_chem_ and T_bio_) have been reported in the scientific literature within the last ~ 10 years. Inspecting the nuclear receptor superfamily the same tendency holds true for 1998–2005 but later the proportion of targets belonging to T_chem_ continuously increased, pointing to investigations on novel targets with potent compounds (but lacking approved drugs), such as investigations on metabolic nuclear receptors^21,22^.

In general, a higher fraction of targets from the categories T_chem_ or T_bio_ in recent years, highlights opportunities for drug discovery that are underexplored. These tendencies are more pronounced for the protein classes of transporters, kinases, and proteases (see Fig. 3). For kinases, even targets of the category T_dark_ in 2008, 2011, and from 2013–2016 (one target in every year) are observed. The highest fractions of T_chem_ and T_bio_ (compared to all other years) can also be attributed to the same years which is the result of large screening studies^18,19^.

Taken together the results from studying the evolution of target trend measures, a shift from GPCR-focused to kinase-focused research can be observed within the last two decades. Results from tracking the target developmental levels suggest that future research should be directed towards unexploited areas in kinase, protease, transporter, and nuclear receptor focused studies since these families are still offering a wealth of targets with no approved drugs. It will however depend on the available research infrastructure, collaborations, and funded projects, if the “hard targets” (e.g. transmembrane transporters) can also be systematically deorphanized in the future. Initiatives like the EU-funded project Resolute^23^ which concentrates on deorphanizing Solute Carriers will hopefully pave the way for other projects uncovering the unexploited opportunities in the human proteome.

### Evolution of gene ontology annotations

Moving from the level of target families to the level of individual targets or target classes enables us to explore biological aspects of target innovation trends encoded in Gene Ontology (GO) annotations. Here, we focus on GO biological process terms as they “represent a specific objective that the organism is genetically programmed to achieve”. Thus, by investigating steep increasing or decreasing trends of certain GO biological process annotations over time, we are able to delineate the evolution of target-driven research interest from a biological perspective. Such trends are identified by fitting a robust regression line to the time evolution of bioactivity counts (counts of measurements) with a certain GO annotation. Bioactivity numbers are expressed as percentages of total bioactivity counts in order to equilibrate the influence of a general upward trend in the number of measurements over time.

Interestingly, when considering only significant trends (p-value ≤ 0.05) the families of kinases and ion channels are showing exclusively positive GO trends (only one significant GO term trend for ion channels with a slope < 0.25), whereas for GPCRs, proteases, NRs, and transporters exclusively negative GO trends were extracted (see Table 1, Supplementary Figs. S1–S6).

**Table 1:** List of annotated GO biological process annotations with positive or negative trends for each target family.

Target family	GO biological process annotation	Slope	Target family	GO biological process annotation	Slope
Kinases	Phosphorylation	2.62	GPCRs	Cell proliferation	− 0.21
	Cellular protein modification process	2.61		Metabolic process	− 0.29
	Signal transduction	2.20		Positive regulation of catalytic activity	− 0.35
	Cell differentiation	1.60		Cell–cell signaling	− 0.63
	Multicellular organismal development	1.56		Circulatory system process	− 0.90
	Response to stress	1.38	Nuclear receptors	Growth	− 0.24
	Immune system process	1.33		Cell differentiation	− 0.24
	Positive regulation of catalytic activity	1.29		Anatomical structure development	− 0.26
	Cell motility	0.93		Reproduction	− 0.27
	Cell death	0.87		Multicellular organismal development	− 0.28
	Cell proliferation	0.71		Biosynthetic process	− 0.50
	Cell cycle	0.66		Cellular nitrogen compound metabolic process	− 0.50
	Anatomical structure development	0.65		Signal transduction	− 0.50
	Cytoskeleton organization	0.63	Proteases	Signal transduction	− 0.23
	Regulation of protein localization	0.50		Multicellular organismal development	− 0.23
	Reproduction	0.48		Cell differentiation	− 0.24
	Cell morphogenesis	0.47		Metabolic process	− 0.25
	Anatomical structure formation involved in morphogenesis	0.47		Cell motility	− 0.28
	Protein complex assembly	0.46		Immune system process	− 0.29
	Biosynthetic process	0.46		Catabolic process	− 0.31
	Homeostatic process	0.42		Response to stress	− 0.32
	Embryo development	0.41		Extracellular matrix organization	− 0.32
	Regulation of catalytic activity	0.39		Proteolysis	− 0.48
	Cell adhesion	0.35	Transporter	Transmembrane transport	− 0.72
	Vesicle-mediated transport	0.35		Transport	− 0.78
	Cell division	0.33			
	Cellular nitrogen compound metabolic process	0.32			
	Positive regulation of apoptotic process	0.31			

The trend is characterized by the slope of the fit (using robust linear regression) of annotation count to year. Only statistically significant trends (p ≤ 0.05) for which the slope ≥  ± 0.25 are shown.

**Figure 4:**
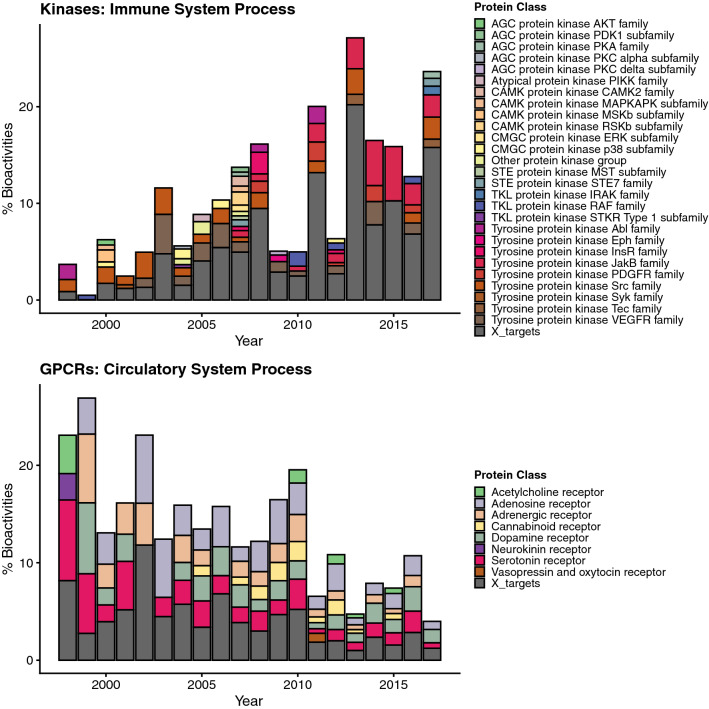
Bar charts showing the proportions of different protein classes contributing to the GO biological process annotation trend “immune system process” for kinases and “circulatory system process” for GPCR targets. Grey bars indicate target classes with little contribution to the general trend (less than 30% of the largest portion of a protein class in the respective year).

In the case of kinases, investigations on targets annotated with “immune system process” (definition: any process involved in the development or functioning of the immune system; see also Supplementary Table S1) have increased during the investigated period (Table 1). Notably, the main contributing class of proteins for this emerging trend are Janus kinases (JAKs) in recent years (Fig. 4). This class of non-receptor tyrosine kinases^24^ are essential mediators for downstream signaling via STAT (signal transducer of activation) proteins and require a soluble factor to associate extracellularly with the corresponding transmembrane receptor for their activation, including cytokines and hormones. Since the JAK/STAT pathway is the major paradigm for membrane to nucleus signaling, it is also recognized as an important regulator of the immune system including the resistance to infection and maintenance of immune tolerance^25,26^. JAKs are therefore targeted by many modern pharmaceuticals for e.g. treatment of autoimmune diseases such as myelofibrosis and rheumatoid arthritis.

While attention in GPCR protein classes involved in “immune system processes” increased since 1998, the research interest in protein classes involved in “circulatory system processes” (definition: organ system process carried out by any of the organs or tissues of the circulatory system) decreased within the same time period for GPCR proteins. Specifically, a shrinking percentage of bioactivity data was reported for dopamine receptors, adrenergic receptors, and adenosine receptors (Fig. 4). Such a trend points to decreasing interest in GPCRs as direct drug targets for cardiovascular diseases. Instead, molecular targets which are part of the signaling pathways that these GPCRs elicit (such as beta-arrestins) are nowadays investigated as promising new avenues for treating heart diseases^27^.

### Evolution of disease annotations

At the level of diseases, target annotations appear to provide even more meaningful insights into target innovation trends since they delineate the extent to which the involvement of a protein (or protein class) in a certain biological process can potentially be translated into the development of pharmaceuticals for the treatment of a particular disease.

With respect to the overall distribution of the trends among the different target families, disease annotations seem to behave similarly to GO biological process annotations: For kinases, exclusively positive trends were found to be statistically significant; for GPCRs, proteases, NRs, and transporters disease trends are exclusively negative (no significant disease trends for ion channels). No particular attempt was undertaken to filter out disease annotations that are describing tightly connected diseases (e.g. “endometrioma” and “endometriosis”) or subcategories of another disease annotation (e.g. “pancreatic neoplasm” and “malignant neoplasm of pancreas”) in order to not bias the trends by individual knowledge about certain diseases.

Across all target families, target classes annotated to different types of cancer are by far the most frequently investigated ones as they show steep increasing trends in research attention over 20 years (Table 2, Supplementary Figs. S7–S11).

**Table 2:** List of annotated diseases (from DisGeNET^34^) with positive or negative trends for each target family.

Target family	Disease	Slope	Target family	Disease	Slope
Kinases	Intellectual disability	0.67	Nuclear receptors	Mood disorders	− 0.26
	Malignant neoplasm of breast	0.44		Anaplastic carcinoma	− 0.27
	Breast carcinoma	0.39		Carcinoma	− 0.27
	Malignant neoplasm of prostate	0.38		Carcinoma, spindle-cell	− 0.27
	Adenocarcinoma of lung (disorder)	0.38		Carcinomatosis	− 0.27
	Adenocarcinoma of large intestine	0.38		Undifferentiated carcinoma	− 0.27
	Prostatic neoplasms	0.37		Animal mammary neoplasms	− 0.28
	Melanoma	0.36		Mammary carcinoma, animal	− 0.28
	Schizophrenia	0.35		Mammary neoplasms, experimental	− 0.28
	Malignant neoplasms	0.33		Adenocarcinoma	− 0.28
	Non-small cell lung carcinoma	0.31		Adenocarcinoma, basal cell	− 0.28
	Mammary neoplasms	0.31		Adenocarcinoma, oxyphilic	− 0.28
	Mammary neoplasms, human	0.30		Adenocarcinoma, tubular	− 0.28
	Colorectal cancer	0.27		Carcinoma, cribriform	− 0.28
	Metastatic melanoma	0.25		Carcinoma, granular cell	− 0.28
Transporter	Major depressive disorder	− 0.26		Breast carcinoma	− 0.31
	Unipolar depression	− 0.26		Mammary neoplasms	− 0.31
	Colorectal cancer	− 0.26		Mammary neoplasms, human	− 0.31
	Rheumatoid arthritis	− 0.26		Malignant neoplasm of breast	− 0.35
	Depressive disorder	− 0.32	GPCRs	Bradycardia	− 0.25
	Mental depression	− 0.32		Major depressive disorder	− 0.26
	Schizophrenia	− 0.33		Mood disorders	− 0.44
Proteases	Liver cirrhosis, experimental	− 0.27			

The trend is characterized by the slope of the fit (using robust linear regression) of annotation count to year. Only statistically significant trends (p ≤ 0.05) for which the slope ≥  ± 0.25 are shown.

**Figure 5:**
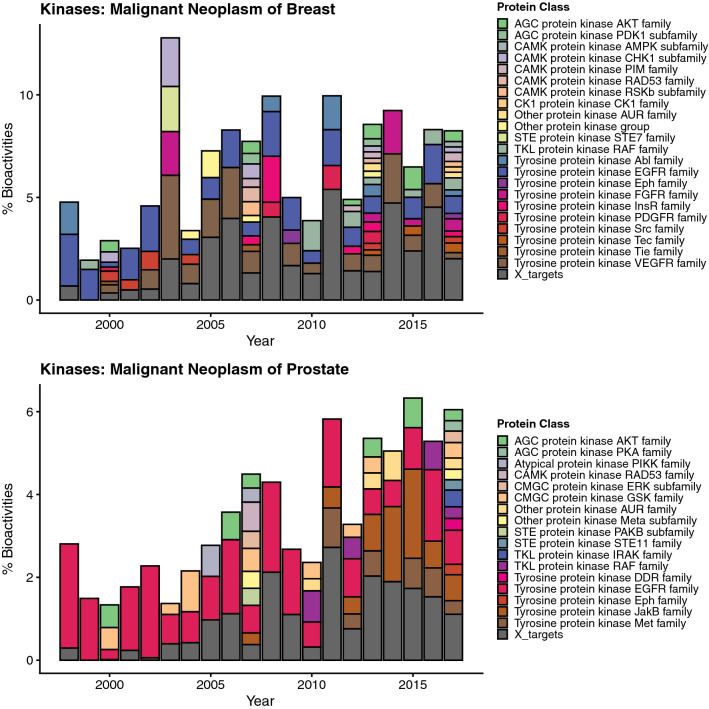
Bar chart showing the proportions of different kinase protein classes contributing to the steep positive disease trends “malignant neoplasm of breast” and “malignant neoplasm of prostate”.

Studying the level of individual protein classes contributing to steep bioactivity trends for cancer-related targets it becomes obvious that research was not focused on only a few protein classes. Instead, depending on cancer type diverging classes of kinases seem to be a focus of interest in recent times (Fig. 5, Supplementary Fig. S12). However, a few protein classes are more frequently investigated across different cancer related disease trends in recent years (Table 2): epidermal growth factor receptor (EGFR) class, vascular endothelial growth factor receptor (VEGFR) class, RAF kinases, and the proto-oncogene MET kinases. Whereas EFGR, VEGFR, and MET are receptor tyrosine kinases (RTKIs) expressed at cell-surfaces, RAF is a downstream kinase transmitting the signal to the cell nucleus. Interest in ligands targeting RAF is a more recent trend (Fig. 5, Supplementary Fig. S12) which reflects emerging approaches in cancer research which are designed to target at the pathway level, ideally by the use of multitargeted TKIs. One prominent example is sorafenib, which inhibits VEGFR, PDGFR- (beta-type platelet-derived growth factor receptor), C-RAF, B-RAF, and c-KIT^28^. A major problem in cancer therapy is the eventual development of resistance to neoplastic therapy, which at the level of targeted anticancer therapy can be attributed to multiple resistance mechanisms including single point mutations, as well as the activation of parallel salvage pathways. Thus, a recent strategy in the development of newer antineoplastic agents is the simultaneous inhibition of multiple pathways. For example CUDC-101^29^ synergistically blocks key regulators of EGFR/HER2 signaling pathways, as well as multiple compensatory pathways, such as AKT, HER3, and MET^30^. The increasing interest in VEGFR as target for antineoplastic drugs is attributed to its effect in preventing tumor-neovascularization (antiangiogenic therapy)^31^.

The JAKB family which has been identified as a main contributor to the steep GO biological process trend “immune system process” for kinases only appeared to contribute to the emerging trend in the case of malignant neoplasm of prostate”, and “adenocarcinoma of large intestine” (Fig. 5, and Supplementary Fig. S12).

Besides cancer-related diseases, “intellectual disability” (subnormal intellectual functioning which originates during the developmental period; previously referred to as “mental retardation”)^32^ and “schizophrenia” are also among the steep positive disease trends for kinases (Table 2, Supplementary Fig. S13). Notably, for nuclear receptors cancer-related diseases are showing negative trends (Table 2, Supplementary Fig. S9). Cancer research thereafter tends to shift to mainly kinase targets while research at nuclear receptor targets in the domain of cancer likely diminished over the past 20 years.

As already apparent from GO biological process annotations, interest in GPCR targets associated to cardiovascular diseases decreased which is confirmed by the declining interest in GPCRs associated with cardiovascular diseases (such as “bradycardia”). In addition, mood disorders seem to be subjected to declining attention in GPCR research (for individual protein class contributions to the negative disease trends for GPCRs see Supplementary Fig. S14).

At the level of all targets (independent from protein classification into target families), derived disease trends can look quite different from those derived for the respective target families (the full list of 237 statistically significant target-based disease trends is provided as Supplementary Table S2). E.g., trends for targets annotated to “schizophrenia” are showing a steep decreasing trend across all targets (Supplementary Fig. S16) while for kinases a slight upward trend was observed. The same tendency holds true for “malignant neoplasm of breast” and other forms of cancer which overall are following a downward trend (Supplementary Fig. S16) while among kinase targets, those annotated to these diseases are increasingly investigated over time. On the other hand, the steep positive disease trend for “intellectual disability” (already observed within the kinase family), was also extracted as the steepest disease trend considering all data (Supplementary Fig. S15). Also, e.g. some forms of cancer are showing moderate positive disease trends overall (Supplementary Fig. S15). These are examples for diseases which are associated to increasingly investigated targets across different target families and which are offering multiple possibilities for interference with small molecules.

### Directing future research

We implemented a web application (https://rguha.shinyapps.io/MovingTargets/), which allows the interactive exploration of target, biological process, and disease trends. From the aggregated visualizations (by protein family, protein class) the individual trends for protein families and target classes can be derived. Links between a disease and GO terms as well as their evolution over time can be visualized by choosing the aggregation by GO biological process.

The target-based approach presented here to identify increasing or decreasing trends in diseases can provide an overview of possible disease-intervention strategies at an early stage in the preclinical drug discovery process. When starting a new drug discovery project, researchers can inform themselves not only about those possible (protein target-based) interventions, but also about the extent of research already available in the domain of certain targets. This can help to take decisions with respect to the importance or relevance of a new project, especially when combining such an analysis with data from clinical study reports. Given the very different time points in the drug discovery process between preclinical literature and clinical reports, using both sorts of information can enable a more robust decision-making process.

To exemplify this approach, we have queried ClinicalTrials.gov (CT)^33^ for reports over time for certain diseases that were listed as showing statistically significant target-based disease trends (considering data for all protein targets; 237 disease trends; Supplementary Table S2). In particular we included multiple synonyms for each disease that was queried (e.g., AML, Acute Myeloid Leukemia, Acute Myeloblastic Leukemia, Acute Non-Lymphocytic Leukemia). Examples are given for acute myeloid leukemia (“Leukemia, Myelocytic, Acute”; slope = 0.29)—a disease associated with a steep positive target trend—as well as for schizophrenia (slope = − 0.90) which is associated with a steep negative target trend (Supplementary Table S2). Intellectual disability was not chosen as an example because it is a higher-level disease term that includes multiple other disease, e.g. Down’s syndrome, fragile X syndrome.

**Figure 6:**
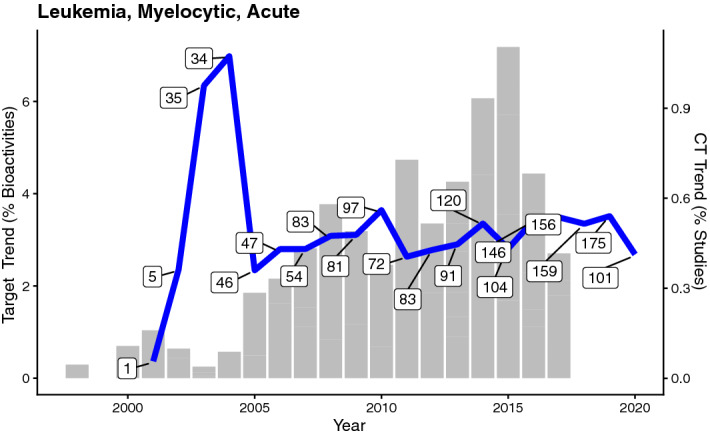
Overlay of target-based disease trend (grey bar chart) and CT-based disease plot (blue line plot) for Acute Myelocytic Leukemia. Target trends are given as percentages of total bioactivity counts, CT trends as percentage of studies. Numbers of studies reported in CT per year are indicated in boxes.

Looking at the overlay of target-based trend plots versus trends retrieved by extracting reports from CT for AML (Fig. 6), one can see that while more research has recently been undertaken on targets related to AML, the trend for studies extracted from CT related to this disease is rather flat since 2005. This could suggest that the preclinical (or research) trend has not yet been translated to the clinical setting and potential worthwhile future clinical studies including heavily investigated current drug-target combinations could be extracted from the target trends. Such an analysis would benefit from text mining approaches in order to make sure that the increasing interest in a target (class) is actually attributed to the disease under study.

**Figure 7:**
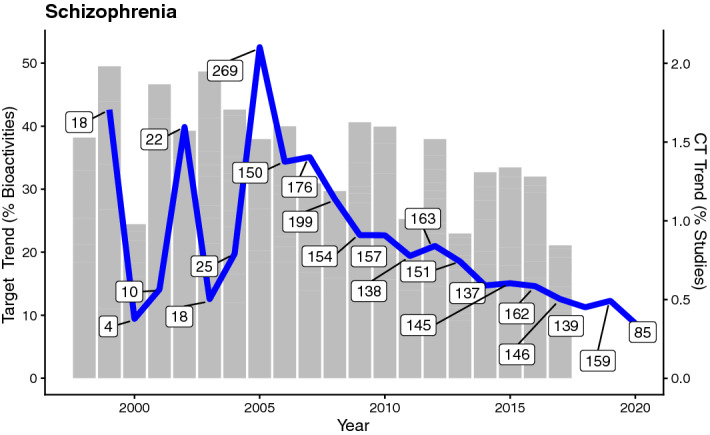
Overlay of target-based disease trend (grey bar chart) and CT-based disease plot (blue line plot) for Schizophrenia. Target trends are given as percentages of total bioactivity counts, CT trends as percentage of studies. Numbers of studies reported in CT per year are indicated in boxes.

The example of schizophrenia (Fig. 7), with a negative trend in both target-based disease trends and CT disease trends, suggests that targets related to this disease are less studied (and therefore have dropped in interest). It might indicate that past efforts invested in studying certain targets were not successful and that, therefore it would not be advisable to spend more resources on them. The downward trend from CT reports related to schizophrenia, also indicates that there is less interest in this disease currently. Again, no causality between the intensity of research in the domain of a target and a disease can be established by this analysis. It can only give an indication for potentially interesting targets to look into.

### Correlating disease trends: Are target trends descriptive of disease trends?

In the previous sections, we have described (1) target families of emerging interest in drug discovery (by tracking target innovation trends comparing different protein families), (2) biological processes of increasing attention (in the form of GO annotations), as well as (3) trends of annotated diseases.

Because disease trends were established by linking diseases to target trends via DisGeNET annotations, the question remains if target trends are at all descriptive of disease trends. We tackled this question by retrieving disease trends from PubMed independent of protein targets and bioactivities and performing a Granger causality test^35^ to the respective target-based disease trends. Since disease trends derived from PubMed are based on publication counts (further referred to as *Dp*) they were correlated to target-based disease trends from our previous analyses by also inspecting paper counts (referred to as *Dt*). Respective trends were derived both by using absolute numbers and by retrieving the normalized paper counts in percent (relating it to the total number of documents per year in PubMed and the dataset extracted from ChEMBL25).

We first considered all the diseases for which we have target-derived trends and retrieved yearly PubMed counts (3253 diseases). For 2041 diseases (for which there was data in at least 10 independent years available in PubMed), we computed the Pearson R^2^and tested for Granger causality between *Dp* and *Dt* for each disease. We then tested for causality in both directions (i.e., is *Dp* causal of *Dt* ?, and vice versa), resulting in two p-values (gt1 and gt2).

**Figure 8:**
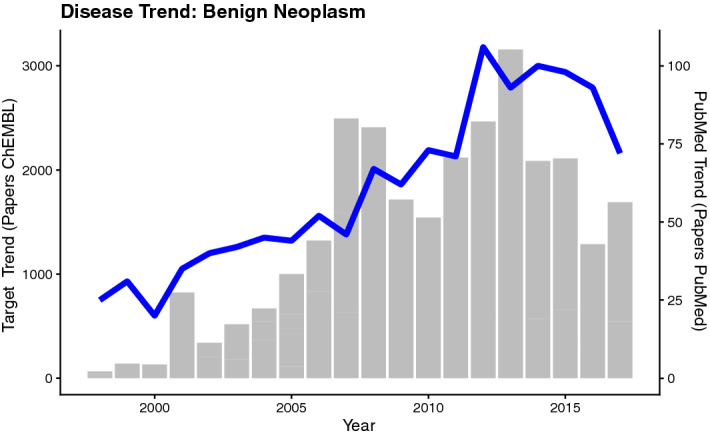
Line plot and bar chart showing the disease trend for “Benign Neoplasm” as expressed by paper counts in PubMed (blue line plot) and ChEMBL (grey bar plot).

Considering the normalized disease trends, there are no diseases for which *Dp* and *Dt* exhibit statistically significant Granger causality (when setting p-value to 0.05). By utilizing the absolute documents counts as a measure, sixteen of the target-derived disease trends are Granger-causal of the PubMed-derived disease trends (gt2  ≤ 0.05; Table 3) and two PubMed-derived disease trends are Granger-causal of target-derived trends (gt1  ≤ 0.05; Table 3). An example for a disease trend showing causality between *Dp* and *Dt* is given in Fig. 8 (for “Benign Neoplasm”).

**Table 3:** List of eighteen diseases showing Granger causality between target-based and PubMed-derived disease trends (p-values gt1 or gt2  ≤ 0.05).

Disease	rare (1/0)	n	R2	gt1	gt2
Penttinen-Aula syndrome	1	18	0.403	0.744	0.039
Benign neoplasm	0	20	0.896	0.456	0.033
Endometrioma	0	20	0.882	0.825	0.033
Chemical and drug induced liver injury	0	20	0.880	0.664	0.050
Hepatic encephalopathy	0	19	0.757	0.262	0.021
Drug allergy	0	20	0.725	0.984	0.023
Pancreatic carcinoma	0	17	0.721	0.724	0.008
Stevens-Johnson syndrome	0	17	0.713	0.293	0.050
Toxic epidermal necrolysis	0	17	0.680	0.303	0.039
Kidney neoplasm	0	20	0.665	0.861	0.033
Upper extremity paresis	0	18	0.607	0.578	0.036
Pigmented basal cell carcinoma	0	19	0.594	0.999	0.001
Papillary craniopharyngioma	0	14	0.571	0.855	0.033
Kleine-Levin syndrome	0	14	0.473	0.349	0.023
Acute kidney insufficiency	0	20	0.472	0.018	0.894
Stable angina	0	20	0.467	0.007	0.678
Nodular glomerulosclerosis	0	20	0.393	0.331	0.044
Cyanosis	0	16	0.141	0.435	0.023

Rare diseases are marked by the annotation “1” (diseases from Orphanet). The number of years for which a publication was reported in PubMed for the particular disease (1998–2017) is given in column “n”. “R^2^”….Pearson correlation coefficient.

On principle, the observed lack of causality between the independently derived disease trends (*Dp* and *Dt*) observed for the remaining 2023 diseases could be a result of imprecise coverage of the measures describing the trends (i.e., document counts from PubMed and/or ChEMBL). Both databases cover a certain part of the scientific literature (with PubMed being the much larger resource containing 30 Mio documents) and both are having their individual foci (PubMed being concentrated on biomedical and life-science literature; ChEMBL focusing on Medicinal Chemistry literature). Furthermore, searches were performed in PubMed, which largely is focused on titles and abstracts (though PubMed Central includes full text articles). The effect of individual coverage of data bases might have also influenced the appearance of the normalized disease trends which likely resulted in the inability to detect causality of these normalized trends.

In addition, it remains elusive if document counts alone can be classified as a useful parameter for describing disease trends since they cannot capture the extent of the testing efforts on certain protein targets (described in individual papers and captured by bioactivity counts).

**Figure 9:**
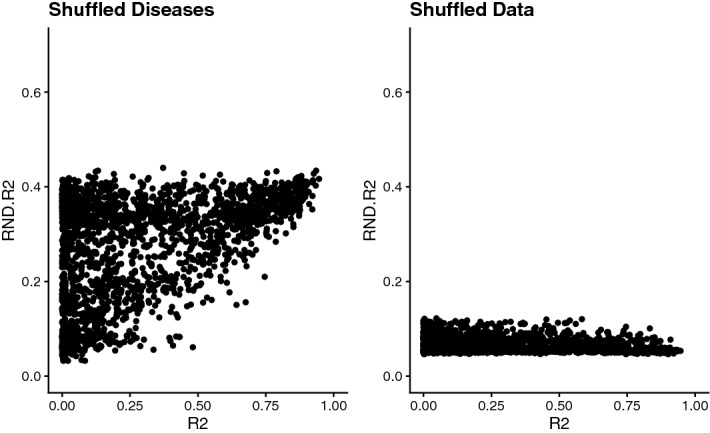
Correlation analyses of original disease trend data from PubMed and randomized data. Pearson correlation coefficients (R^2^) of target-based disease trends are plotted against R^2^ of PubMed-derived disease trends (RND.R^2^) after shuffling diseases (left plot) or after shuffling data points (right plot).

Despite a lack of causality for most disease trends, 22% (453 out of 2,041) of the diseases are possessing a Pearson correlation coefficient of > 0.6 between PubMed and target-derived disease trends (the full list of diseases with R^2^ and p-values for Granger causality test between *Dt* and *Dp* is given as a Supplement). To test whether this is only a random finding, we shuffled the trend data for *Dp,* computed R^2^ values for the randomized data and compared the distribution of R^2^ values of the original and randomized data sets. We shuffled the data in two different ways, once by computing the mean R^2^ of *Dt* to *Dp* of 500 randomly selected diseases, another time by shuffling data points in the respective *Dp* trends of the original diseases a thousand times and as well computing the mean R^2^ to *Dt*. Whereas for the “data shuffling approach” mean correlation coefficients between *Dt* and *Dp* of the randomized data sets (RND. R^2^) are always very low (< 0.13), for the “disease shuffling approach” RND. R^2^ can take values up to 0.44 (Fig. 9). It was also observed that for very small R^2^ values when correlating the original data sets, the latter approach in some cases can give higher mean correlation coefficients after shuffling diseases (but still always below random correlation).

Summarizing the observations on correlation and causality of disease trends, we found that for a fair amount of diseases (22%) trends derived from PubMed publications and disease trends derived from target counts in ChEMBL correspond well. For some diseases, target-derived disease trends are even Granger-causal of PubMed-derived trends. Since certain factors limit this kind of analysis (especially with respect to data completeness), it remains inconclusive if a disease trend analysis merely based on protein targets entirely reflects research attention in a certain area of biomedical research.

## Summary and conclusions

In this study, different views of target innovation are discussed by studying time trends in research attention to particular targets in a protein family-wise fashion. Our study of emerging target families (in terms of numbers of bioactivities, targets, annotated drugs, and papers) highlights kinases and GPCRs as the most heavily investigated protein families. Interestingly, kinases have outpaced GPCRs in recent years which is especially pronounced in terms of investigated compounds and targets. A temporal view of the evolution of target developmental level (TDL) categories reveals areas of interest in target space that have just emerged in the scientific literature, such as T_bio_ and T_dark_ targets, where no active molecules are known. Many targets within these categories are kinases and transporters, highlighting novel opportunities for drug intervention.

Two orthogonal views of target innovation are considered by investigating trends in GO biological process terms and disease annotations over time. We note that no proof can be provided whether there is a causal link between attention being paid to a target or target family and a detected emerging disease or biological process trend. We investigated how intense a particular target was investigated and reported in the medicinal chemistry literature without extracting information on potential indication areas from the respective literature sources. Thus, the emerging biological process and disease trends rather highlight opportunities for therapeutic intervention rather than a real current focus on a particular disease or biological process.

The value of the approach lies in the users’ ability to capture trends in research attention at an early stage, even for researchers who are not experts in a particular research domain.

Importantly, the described methodology to analyze and visualize the data can only depict information that is already available. Its strength lies in the interconnection of the disparate scientific sub-domains: drug discovery and biology linked by bioinformatics analysis. This implies that the method works well when considering data-rich domains, as underrepresented targets families will tend to be ignored if one considers only the number of measurements. However, it can in principle also be applied to only a specific target family or target class in order to identify emerging trends within that particular domain.

One limitation is the availability of GO and disease annotations for particular targets which might be unevenly distributed, biasing the amount of available annotations to already heavily studied targets. An extreme example are orphan targets, where no potent compounds are known and there are few if any diseases or biological process associations.

The question if such an analysis rather contributes to a reinforcement in the study of already heavily investigated targets and diseases—rather than inspiring research on novel targets and in new therapeutic areas—remains. Most likely, the relationships between compounds, biological processes and diseases will deliver the most useful information at the intermediate stage of developmental level of a target. For those cases, a link between the target and a disease has already been demonstrated by the available information, but drug discovery for that particular target or for the envisaged therapeutic indication should ideally be in its childhood still.

Thus—if used in a comprehensive manner—analyzing the temporal “movement of targets” from a data science perspective enables researchers to look back on their way forward when planning and directing their future research. Interactive exploration of target, biological process, and disease trends is possible via an interactive web application available at https://rguha.shinyapps.io/MovingTargets/.

## Methods

### Data retrieval

Data was retrieved from ChEMBL25^36^ via a PostgreSQL interface (pgAdmin4) by querying for compounds along with their bioactivity information (activity type, activity standard value, standard unit etc.), structural information (canonical smiles, standard InChI, and InChIKeys), target information (including protein classification), assay information and document information. In this initial query, filters for the target organism (“Homo sapiens”), target type (“SINGLE PROTEIN”), assay confidence score (> 8), document year (> 1980), and standard units for activities (“NOT NULL”) were set. This led to an initial dataset with 1,213,906 data entries (359,796 unique compounds).

Next, the data file was imported into a KNIME (version 3.5)^37^ environment to perform data filtering, processing and analysis (details are provided in the following subsections).

### Data filtering

The raw data (ca. 1.2 Mio data points) was filtered for publication years 1998–2017. This led to a final data set of 1,198,958 data points (353,784 unique compounds).

### Retrieving and mapping of gene ontology annotations

A list of all protein targets with their respective protein classification as well as GO annotations was retrieved in a separate sql query (data set with 153,000 data points). Subsequent mapping of target ID’s (via the ChEMBL target tid’s) to protein classes and GO annotations was performed in KNIME. GO annotations were filtered for “GO biological process” annotations only.

### Filtering for individual protein families

The data set containing mappings for targets, GO terms, and diseases was split into six major protein classes by filtering the column “protein_class_desc” (protein class description) for *kinase*, *7tm1*, *transcription factor*, *protease*, *ion channel*, *transporter* (to filter for kinases, GPCRs, nuclear receptors (NR), proteases, ion channels, and transporter, respectively). Mapping these six subsets with the large data set (ca. 1,199 K data points) via tid’s led to the final data sets for the six target classes: approximately 400 K data points for kinases, 246 K for GPCRs, 55 K for NR, 68 K for proteases, 44 K for ion channels, and 42 K for transporter. In a separate step, tid—UniProt^38^ ID mappings were retrieved in a sql query and UniProt IDs were added to the final data sets for the six target classes.

### Retrieving drug-efficacy target annotations

ChEMBL provides an annotation, called DISEASE_EFFICACY, which flags whether the target assigned is believed to play a role in the efficacy of the drug in the indication(s) for which it is approved (1 = yes, 0 = no). These annotations were retrieved from ChEMBL via a separate sql query and mapped via target tid’s and drug molregno’s.

### Mapping target developmental level (TDL) categories

Mappings of ChEMBL protein targets to TDL categories were kindly provided by Vishal Siramshetty (NCATS/NIH).

### Retrieving and mapping of disease annotations

Curated gene-disease associations (81,746) and mappings of UniProt IDs and gene IDs were downloaded in CSV format from DisGeNET and imported into KNIME. These gene-disease annotations were collected from UniProt, CGI^39^, ClinGen^40^, Genomics England (https://panelapp.genomicsengland.co.uk/), CTD (human subset)^41^, PsyGeNET^42^, and Orphanet (https://www.orpha.net/consor/cgi-bin/) and provide a score to rank these associations by taking the number and type of sources into account (level of curation, organisms), and the number of publications supporting the association. Only associations with a score equal or greater than 0.3 were retained (at least 1 curated source reporting the gene-disease association; 81,386 associations). First, UniProt IDs were mapped to diseases via the associated gene IDs in KNIME. Then, UniProt IDs served to map diseases to the six target class data sets.

### Trend analyses

Data processing for subsequent trend calculation was performed in KNIME. Target innovation trends, GO term trends, and disease trends were described by a robust linear regression using time (Year) as an explanatory variable and the numbers of published bioactivities (measurements) per year as the response variable. The number of bioactivities was always related to the total number of bioactivities in the respective year, expressing it as a percentage of (total) bioactivities. Trend analyses were performed in R version 3.5.2 using the rlm function (“MASS” package version 7.3–51.1). Statistical significance of the trends was assessed by conducting a Wald test (“sfsmisc” package version 1.1–3) on the fitted model and extracting the p-value. Regression fits with p-values ≤ 0.05 were considered significant, otherwise insignificant.

### Retrieving disease trends from ClinicalTrails.gov

Disease trends as reported in ClinicalTrials.gov were accessed via AACT (Access to Aggregate Content of ClinicalTrials.gov; version aact_20200901), a publicly available cloud-based PostgreSQL database that contains all information (protocol and result data elements) about every study registered in ClinicalTrials.gov (https://clinicaltrials.gov/). The respective data for a given disease was extracted via a PostgreSQL interface (pgAdmin4) by querying for clinical study identifiers (NCT number), the first posted study dates, and the respective conditions (diseases). In order to avoid that data is missed out due to the use of disease synonyms, we looked up possible synonyms (disease mappings) in DisGeNET and queried for all synonyms. Extracted CSV files were imported into KNIME and grouped by study year while counting the number of reports per year for a certain condition (disease). Normalizing the counts to the total numbers of reports per every year, gave a percentage of reports/year for a certain disease which was subsequently displayed in line plots.

### Retrieving disease trends from PubMed

For each disease we queried PubMed using the Entrez API. We only queried for the given disease names and did not consider synonyms or other forms of the name. It should be noted that for multi-word disease names, PubMed automatically translates the query to multiple terms, which can result in false positives. As a result, we ignored the count of articles for the translated query and restricted ourselves to article counts for the disease name as is. Counts were retrieved on a yearly basis. In addition, we retrieved the total number of articles on a yearly basis to enable normalization of per-disease article counts.

### Correlating disease trends

We define a disease trend derived from Pubmed publication counts as *Dp* and a disease trend derived from the target publication counts as *Dt*. Trends are defined in terms of publication counts. For *Dp*, this is the number of publications for that disease name per year as retrieved from PubMed. For *Dt*, this is the sum of the publication counts associated with the targets (as recorded in ChEMBL) for the disease. Trends are compared in terms of absolute document counts (per year) and in terms of normalized (to the total number of documents published that year) document counts (per year). Pearson R^2^ was computed and Granger causality between *Dp* and *Dt* was tested for each disease (by applying the “grangertest” function from the lmtest package in R). For the latter, we test for causality in both directions (i.e., *Dp* is causal of *Dt* and vice versa), resulting in two p-values. The level of significance was set to 0.05.

### Data shuffling

For the correlation analysis between disease trends derived independently from PubMed and ChEMBL data, PubMed trend data was shuffled in two different ways: (1) yearly document counts were shuffled a thousand times for the equivalent disease trend data from PubMed; (2) disease trend data from PubMed was shuffled as a whole by selecting 500 diseases randomly and computing the R^2^ to these PubMed-derived disease trends for each target-derived disease trend. Randomized correlation coefficients (“RND.R^2^”) were computed by retrieving the mean values for the iteratively retrieved R^2^.

### Data visualization

All plots and bar charts were produced in R by using the package “ggplot2” (version 3.1.0).

The data for creating the stacked bar charts showing the proportions of different target classes contributing to a particular GO biological process or disease was prepared in KNIME beforehand. Only protein classes are shown as separate blocks if their percentage of bioactivities in a particular year is at least 30% of the largest contributing protein class (in that year). Target classes with minor contribution to a trend are denoted as “X_targets” in the visuals.

### Data, code, and workflow availability & web application

All data sets, R code, as well as KNIME workflows used for performing the analyses are publicly available: https://github.com/BZdrazil/Moving_Targets. A web application for interactive exploration of target, biological process, and disease trends was developed in “R Shiny” and is available at the following URL: https://rguha.shinyapps.io/MovingTargets/.

## Supplementary information

Supplementary Information 1.

Supplementary Information 2.
