# Supplementary material for: Moving targets in drug discovery
Source: Sci Rep. 2020 Nov 19;10:20213. doi: 10.1038/s41598-020-77033-x (PMC7677539; doi:10.1038/s41598-020-77033-x)
Supplement: Supplementary file 2 — Supplementary Information 2. [file 41598_2020_77033_MOESM2_ESM.pdf]

<u>Supplementary Information:</u>

Moving Targets in Drug Discovery

Barbara Zdrazil,[1,] Lars Richter,[1] Nathan Brown,[2] and Rajarshi Guha[3]*

[1] University of Vienna, Department of Pharmaceutical Chemistry, Division of Drug Design and Medicinal Chemistry, Althanstrasse 14, 1090 Vienna, Austria

[2] BenevolentAI, 4-8 Maple Street, London, W1T 5HD, United Kingdom

[3] Vertex Pharmaceuticals, 50 Northern Avenue, Boston, MA 02210, United States

*Corresponding author: barbara.zdrazil@univie.ac.at

Included Supplementary Figures/Tables:

Supplementary Figure S1-S16
Supplementary Table S1-S2

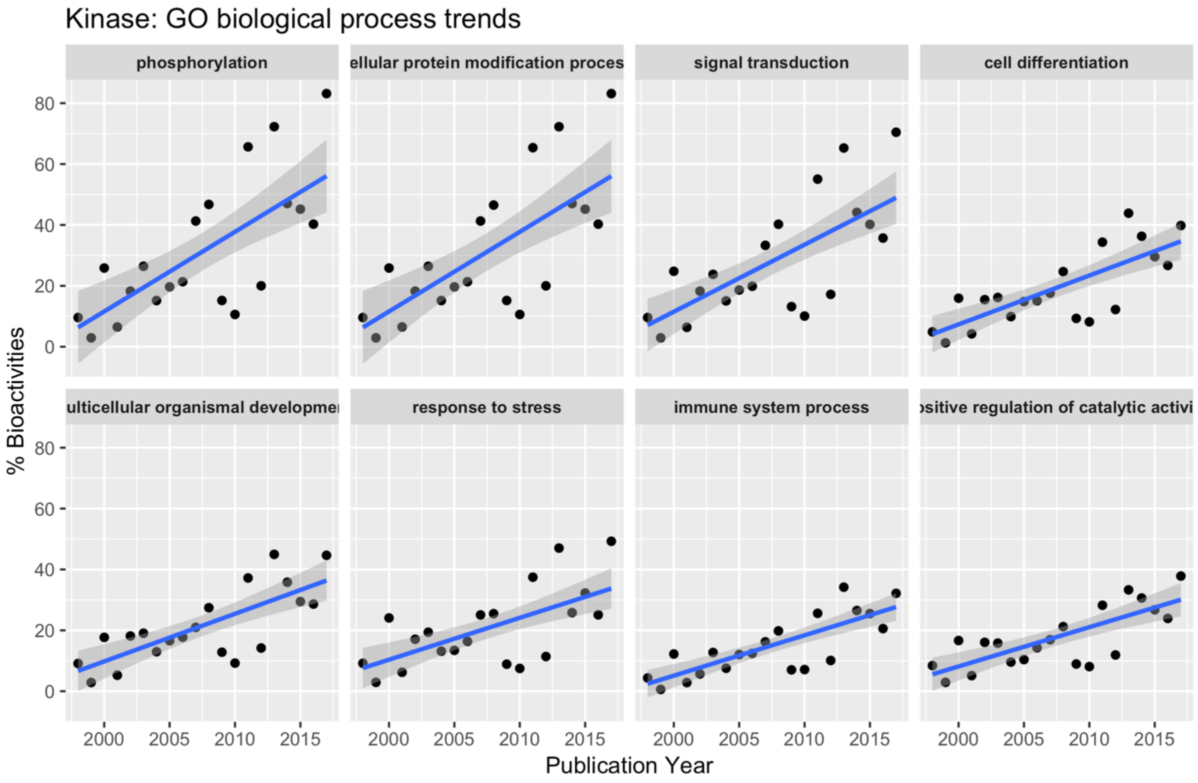

Supplementary Figure S1: *Trend line plots for GO biological process annotations for kinases. Only the eight steepest trends are shown (if available) considering only statistically significant trends (robust regression; $p < 0.05$).*

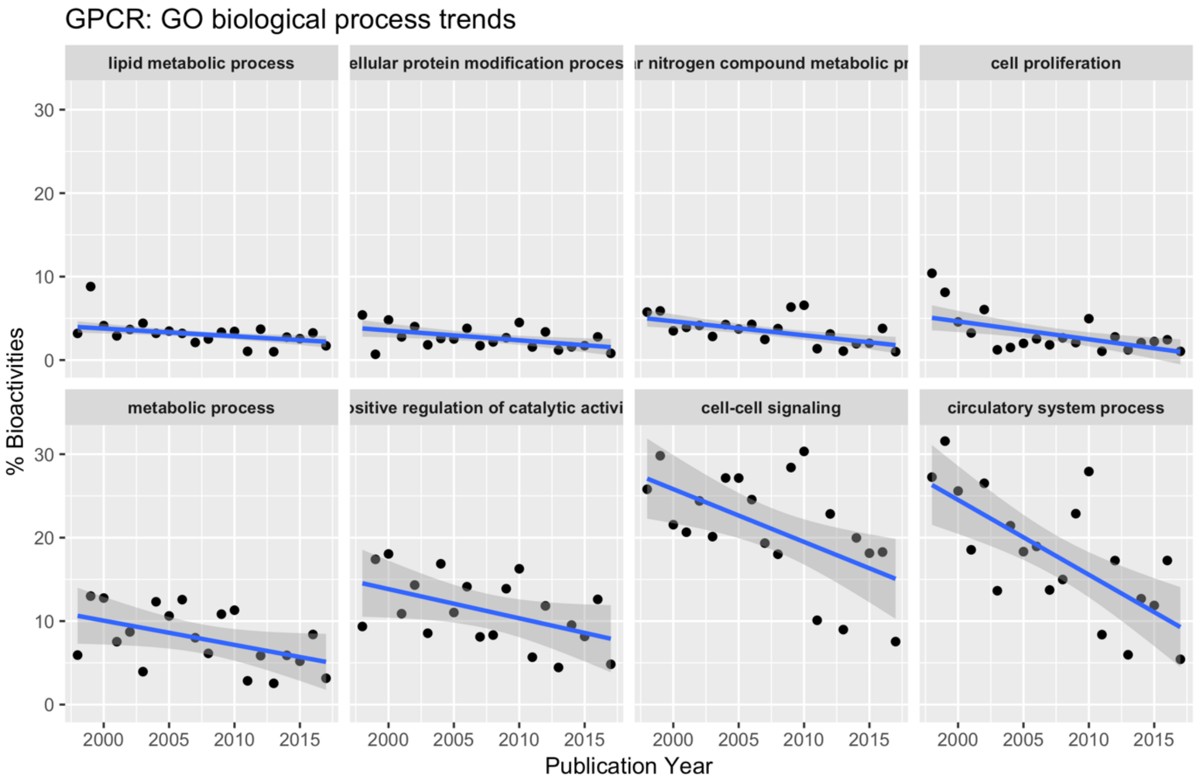

Supplementary Figure S2: *Trend line plots for GO biological process annotations for GPCRs. Only the eight steepest trends are shown (if available) considering only statistically significant trends (robust regression; p < 0.05).*

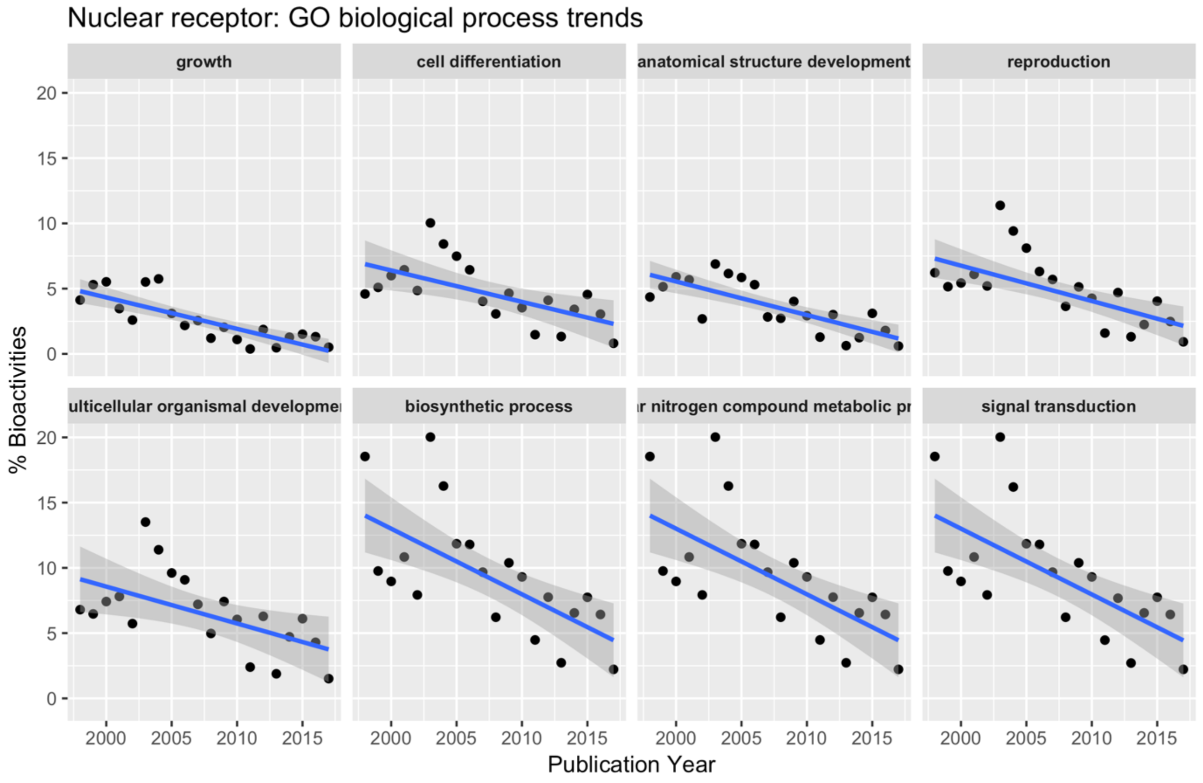

Supplementary Figure S3: *Trend line plots for GO biological process annotations for nuclear receptors. Only the eight steepest trends are shown (if available) considering only statistically significant trends (robust regression; $p < 0.05$).*

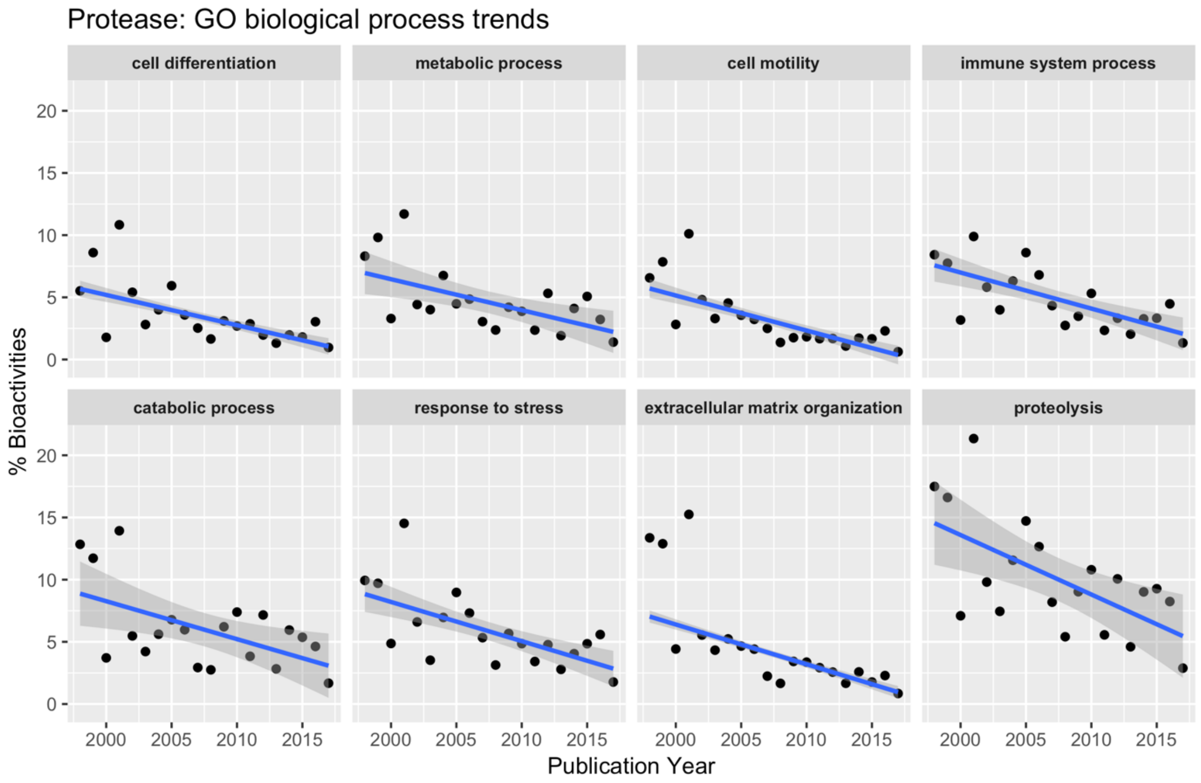

Supplementary Figure S4: *Trend line plots for GO biological process annotations for proteases. Only the eight steepest trends are shown (if available) considering only statistically significant trends (robust regression; p < 0.05).*

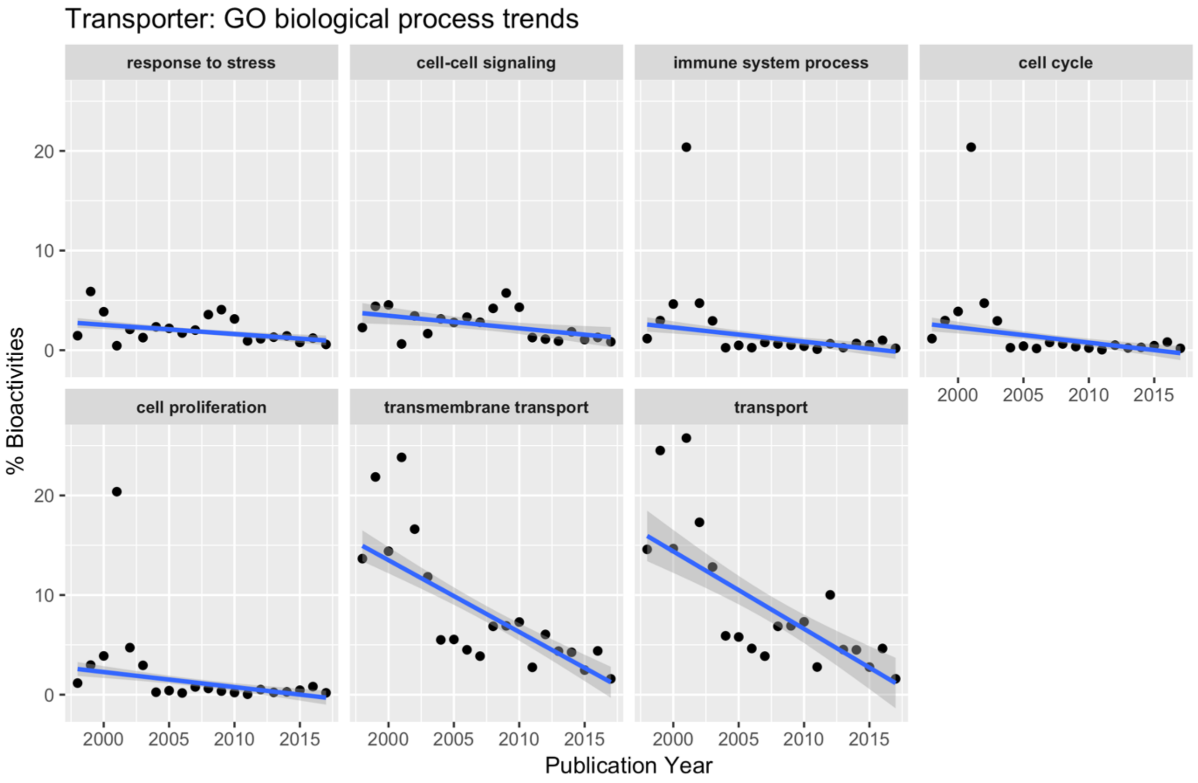

Supplementary Figure S5: *Trend line plots for GO biological process annotations for transporter. Only the eight steepest trends are shown (if available) considering only statistically significant trends (robust regression; $p < 0.05$).*

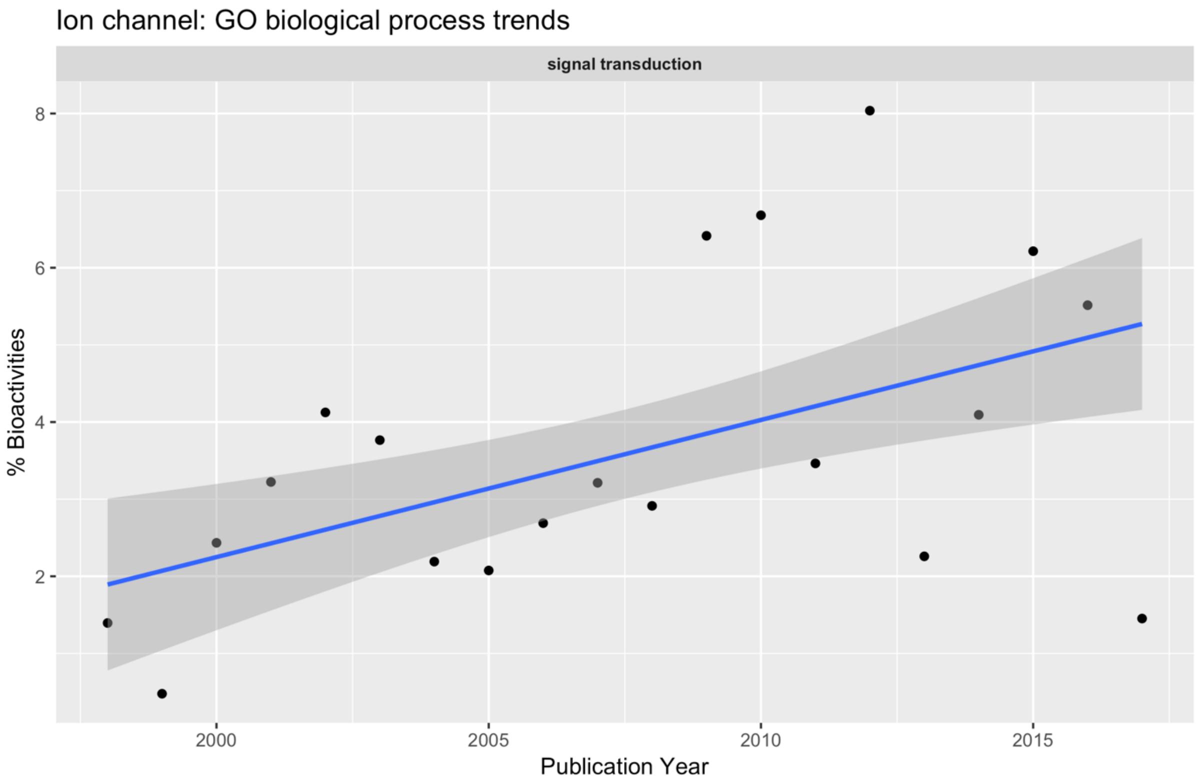

Supplementary Figure S6: *Trend line plots for GO biological process annotations for ion channels. Only the eight steepest trends are shown (if available) considering only statistically significant trends (robust regression; p < 0.05).*

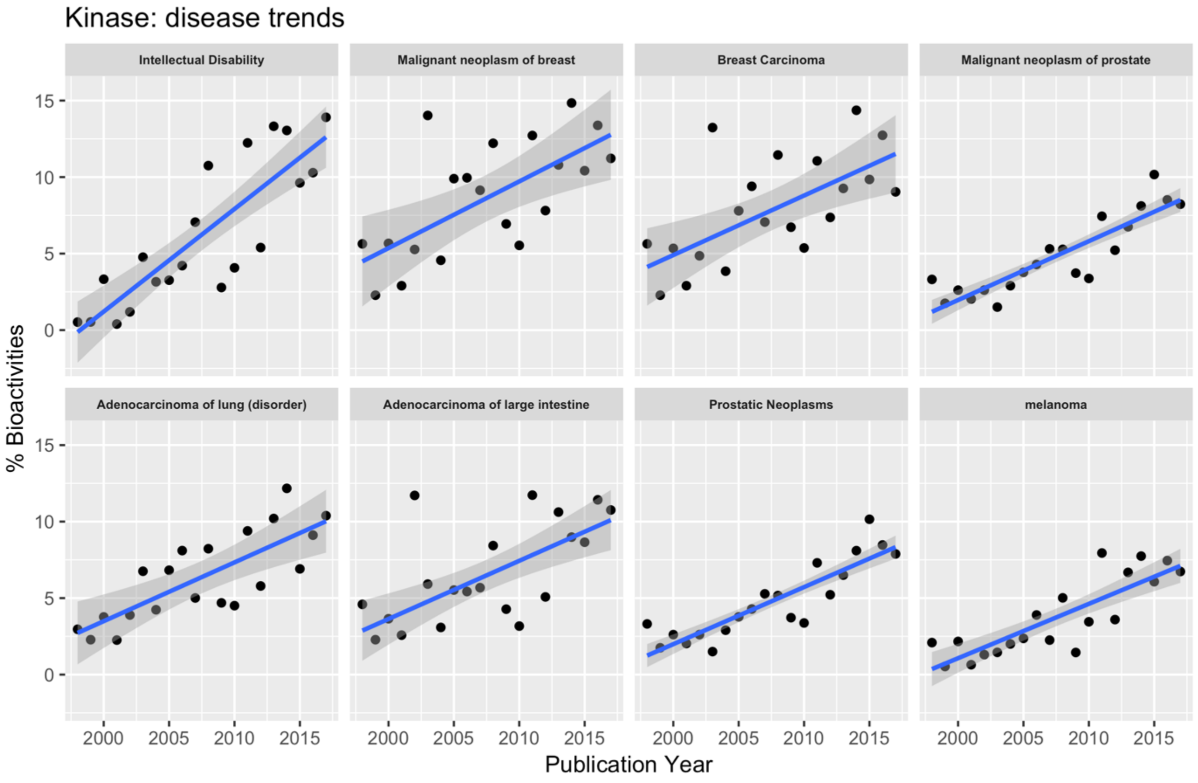

Supplementary Figure S7: *Trend line plots for disease annotations for kinases. Only the eight steepest trends are shown (if available) considering only statistically significant trends (robust regression; $p < 0.05$).*

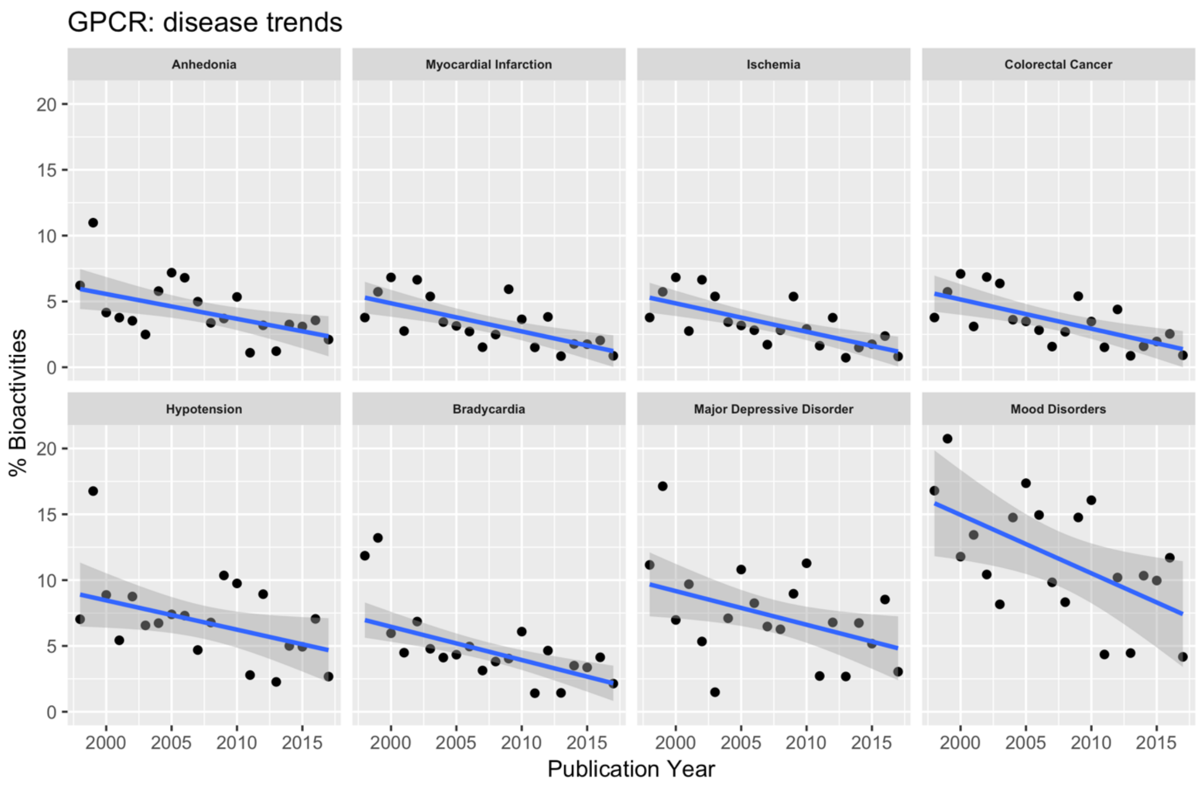

Supplementary Figure S8: *Trend line plots for disease annotations for GPCRs. Only the eight steepest trends are shown (if available) considering only statistically significant trends (robust regression; $p < 0.05$).*

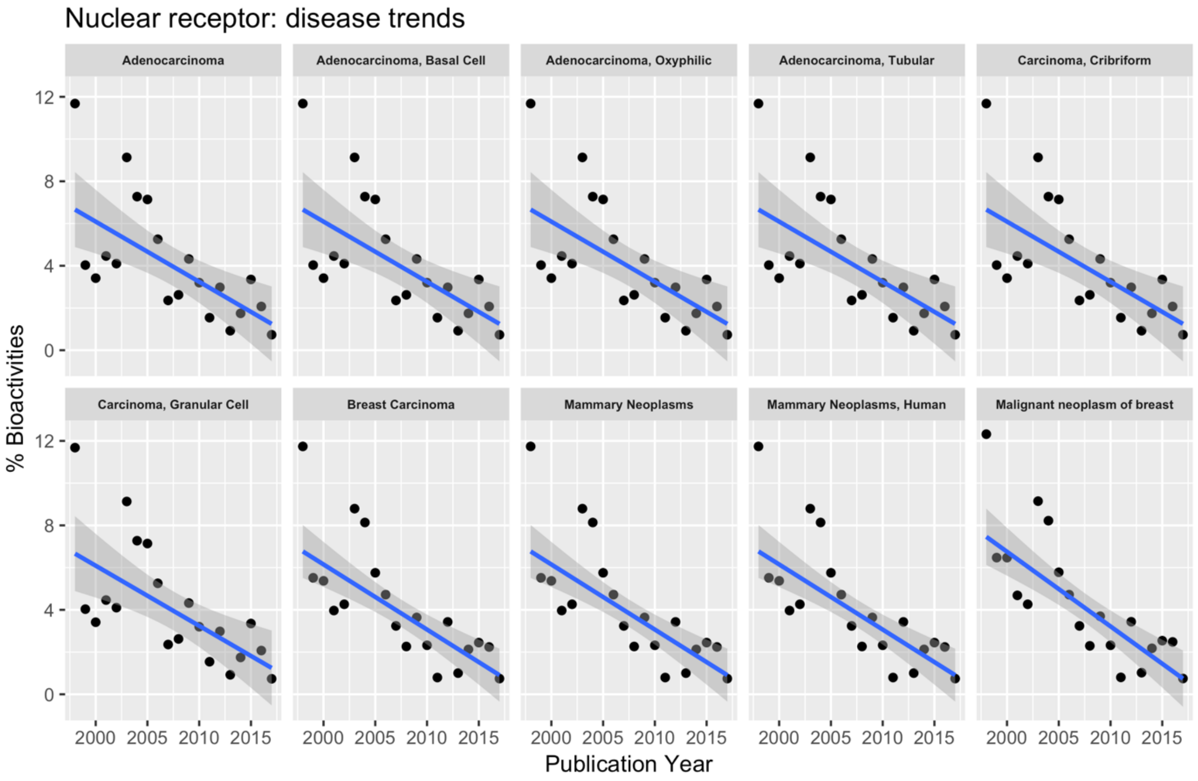

Supplementary Figure S9: *Trend line plots for disease annotations for nuclear receptors. Only the eight steepest trends are shown (if available) considering only statistically significant trends (robust regression; p < 0.05). Because multiple trends among the top eight are having the exact same coefficient for the slope, they are all displayed.*

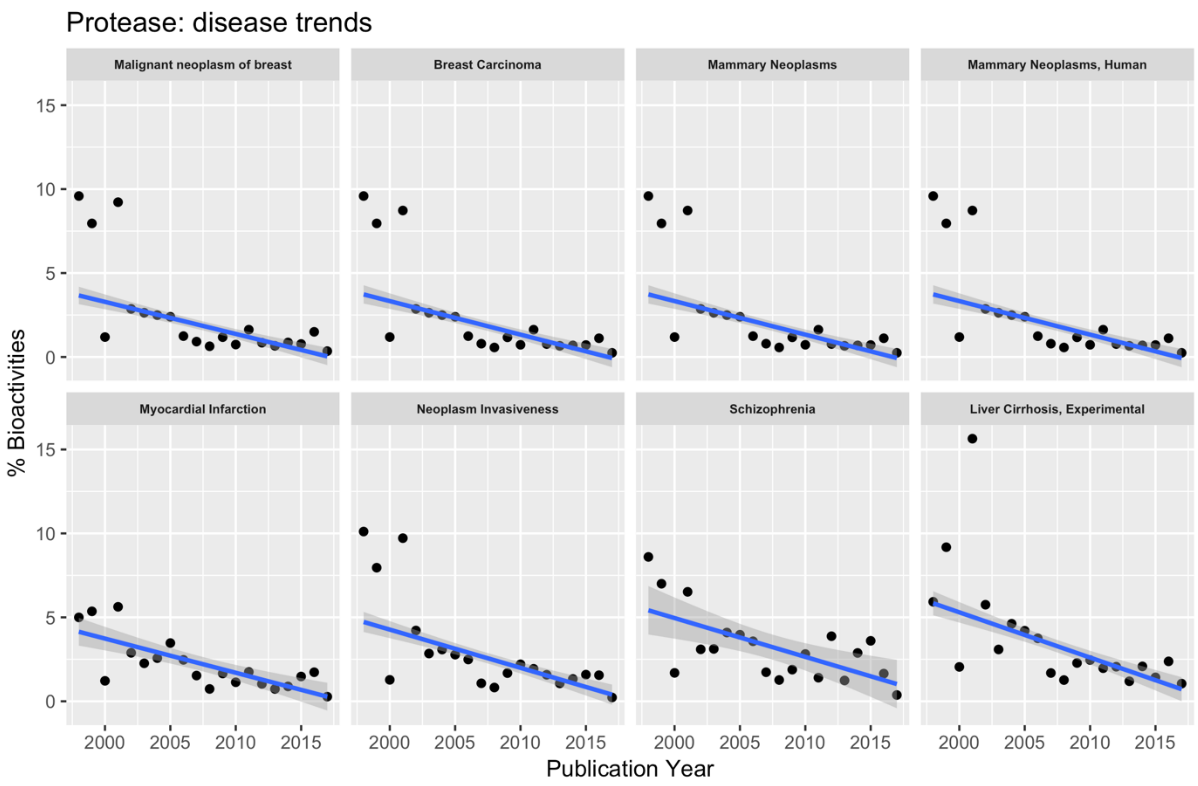

Supplementary Figure S10: *Trend line plots for disease annotations for proteases. Only the eight steepest trends are shown (if available) considering only statistically significant trends (robust regression; $p < 0.05$).*

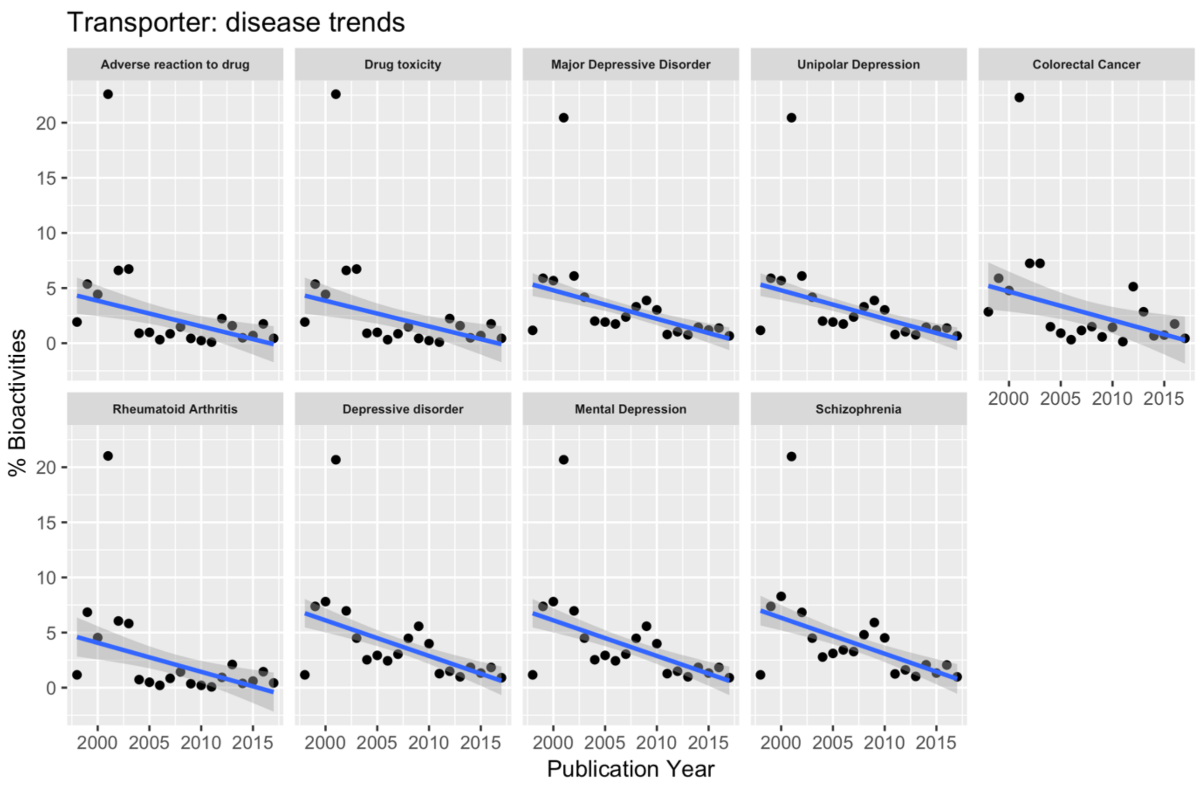

Supplementary Figure S11: *Trend line plots for disease annotations for transporter. Only the eight steepest trends are shown (if available) considering only statistically significant trends (robust regression; $p < 0.05$). Because multiple trends among the top eight are having the exact same coefficient for the slope, they are all displayed.*

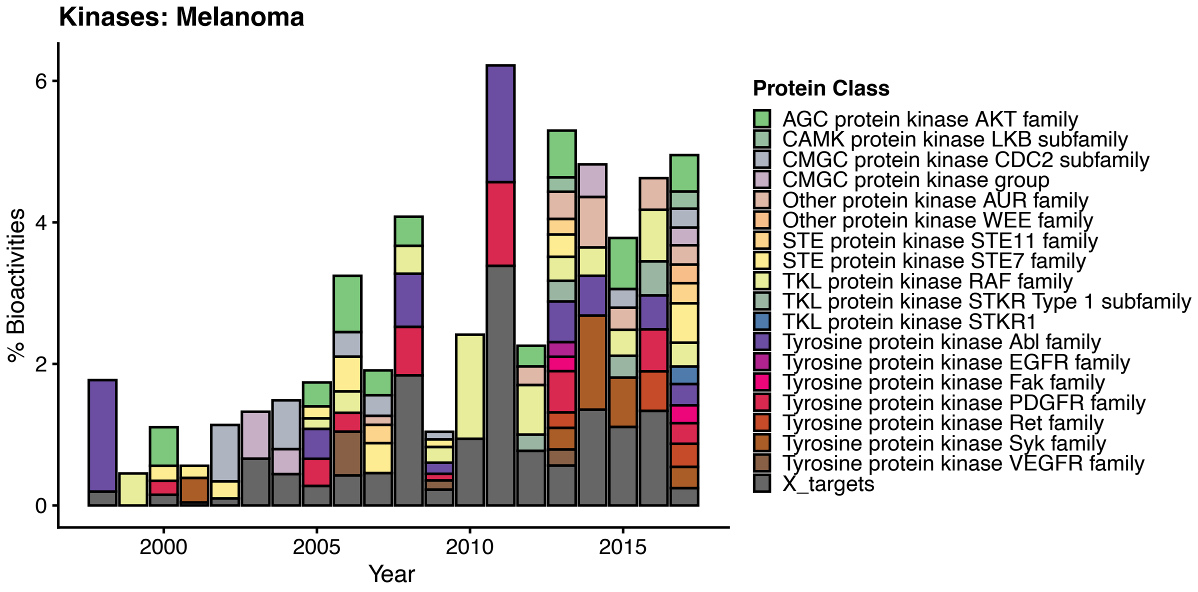

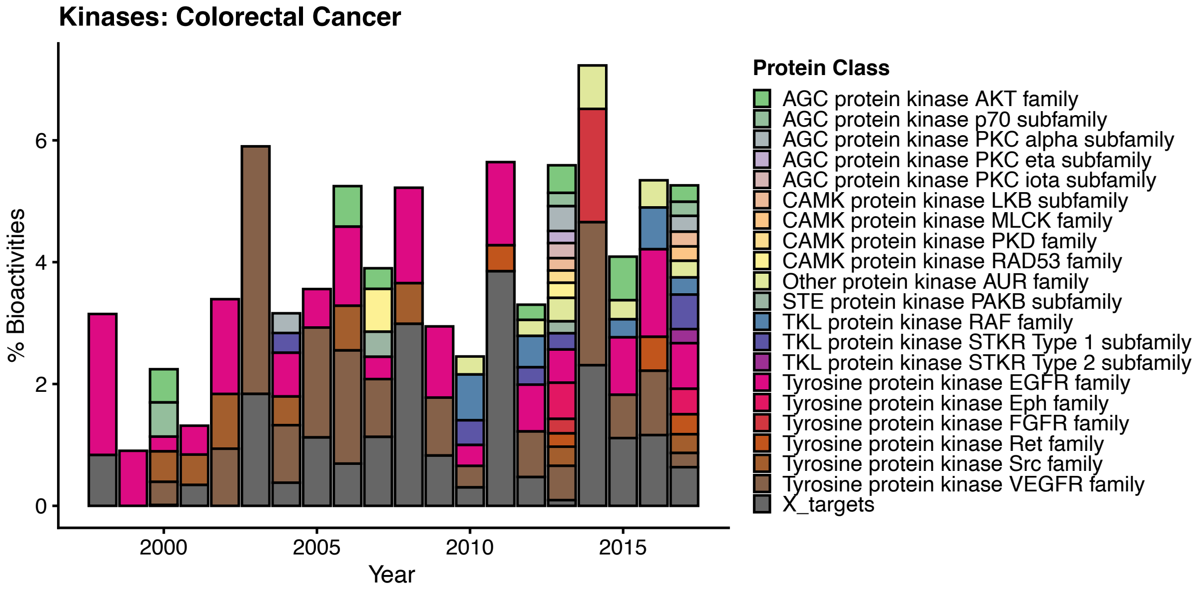

Supplementary Figure S12: *Bar charts showing the proportions of different kinase protein classes contributing to selected steep cancer-related disease trends for kinases.*

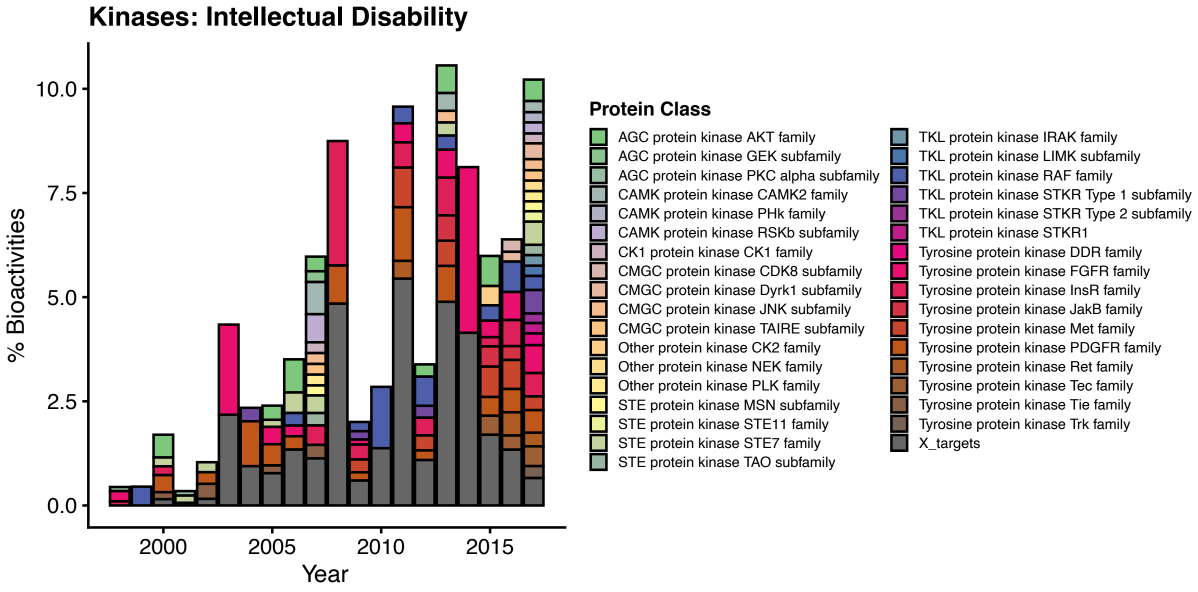

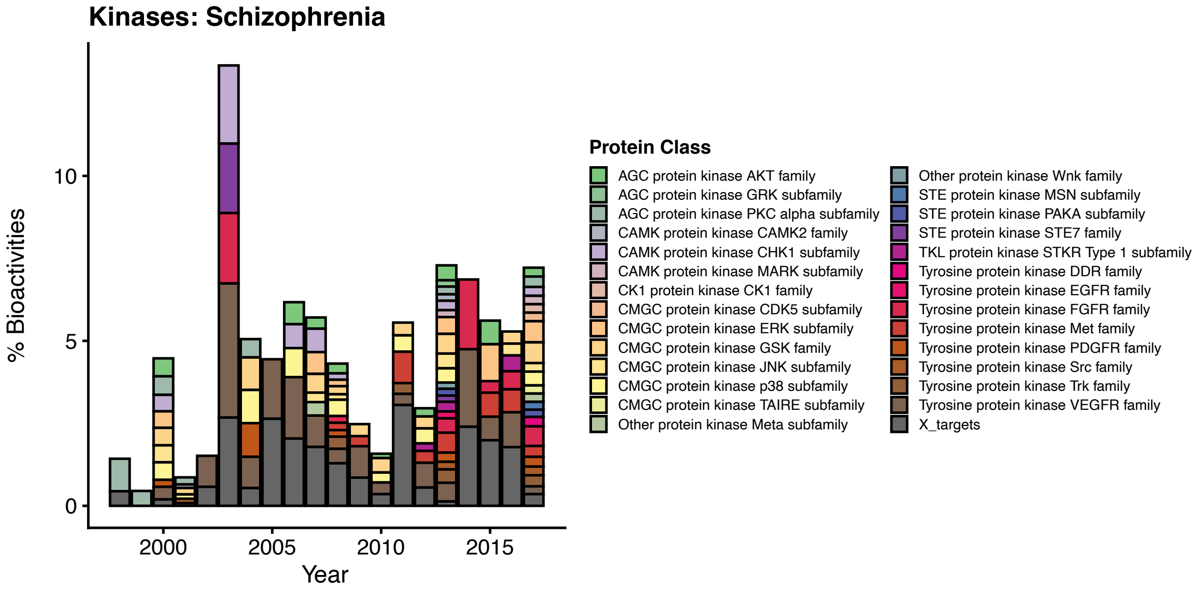

Supplementary Figure S13: *Bar chart showing the proportions of different kinase protein classes contributing to the steep positive disease trends "Intellectual Disability" and "Schizophrenia".*

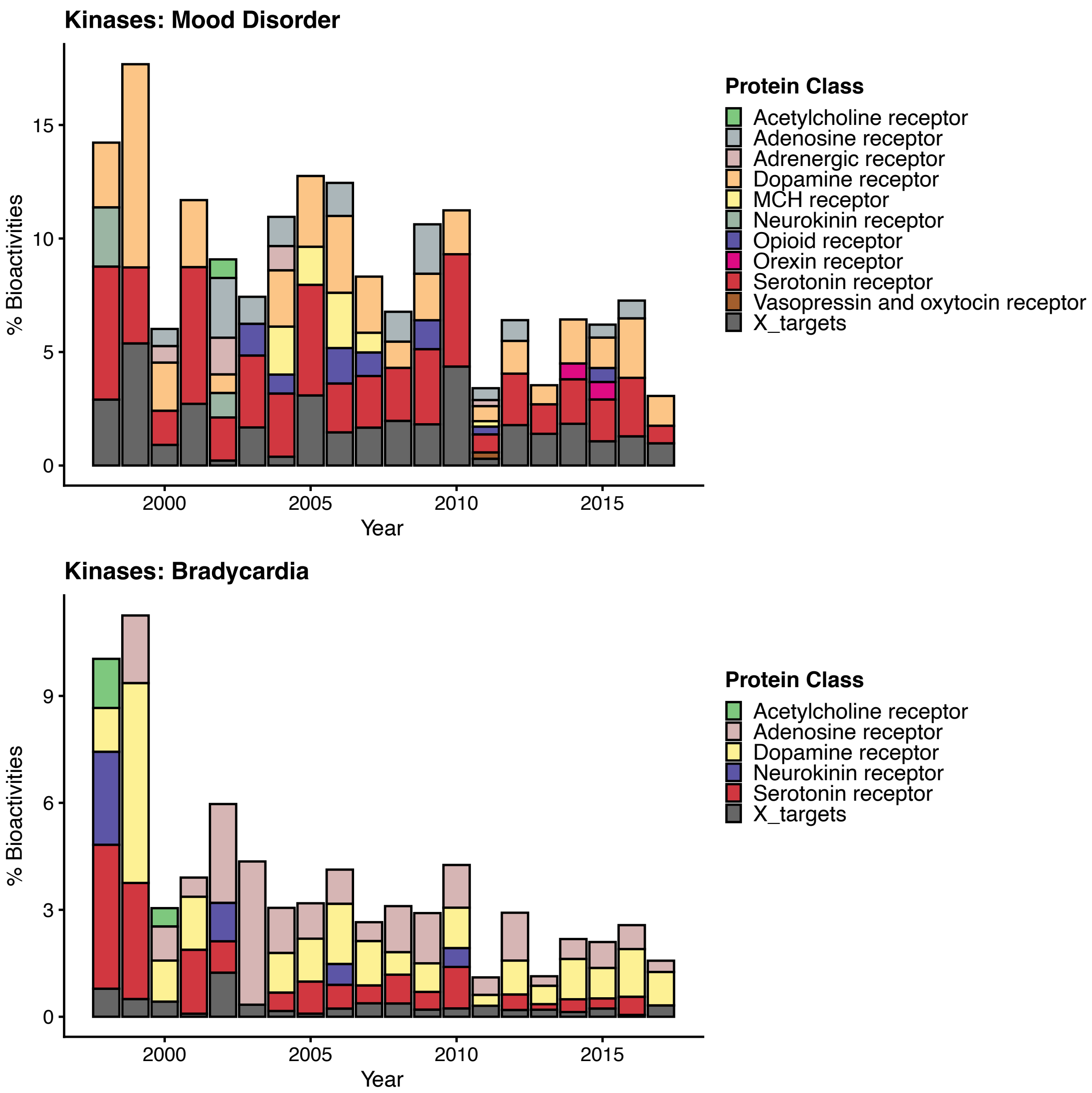

Supplementary Figure S14: *Bar charts showing the proportions of different GPCR protein classes contributing to the steep negative disease trends "Mood Disorders" and "Bradycardia".*

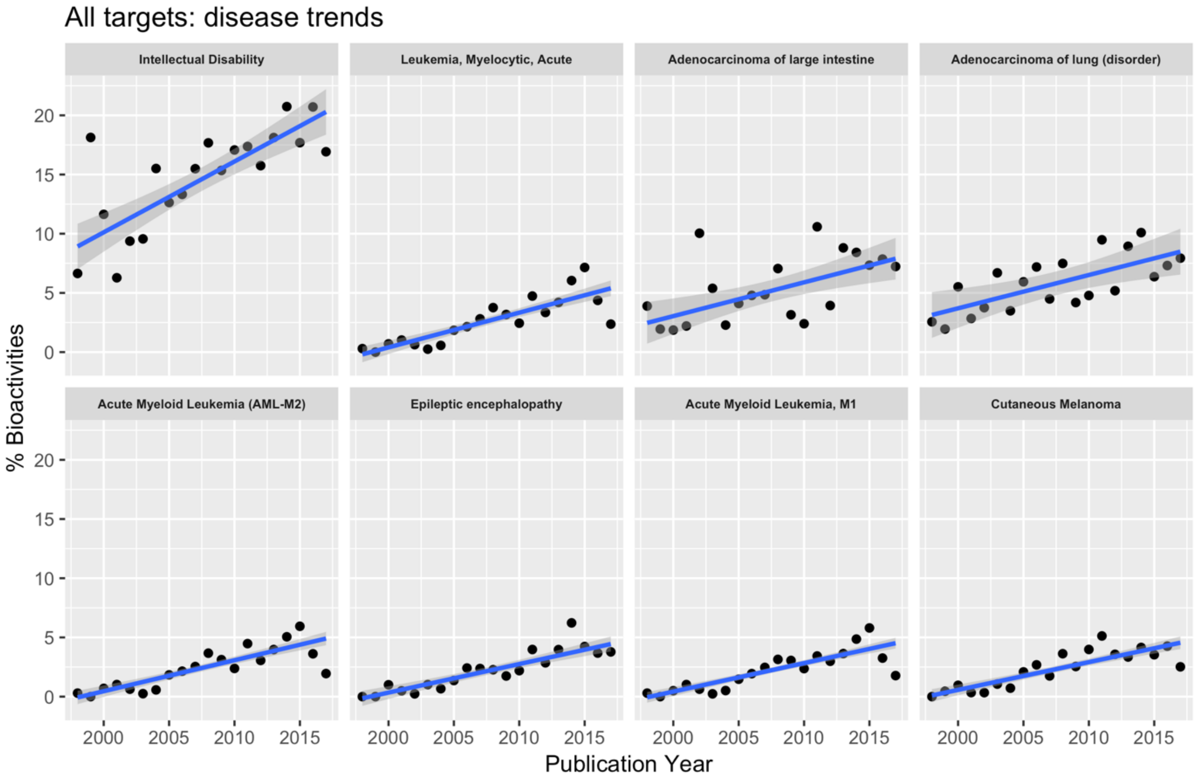

Supplementary Figure S15: *Trend line plots for disease annotations (for the whole data set). Only the eight steepest positive disease trends are shown considering only statistically significant trends (robust regression; $p < 0.05$).*

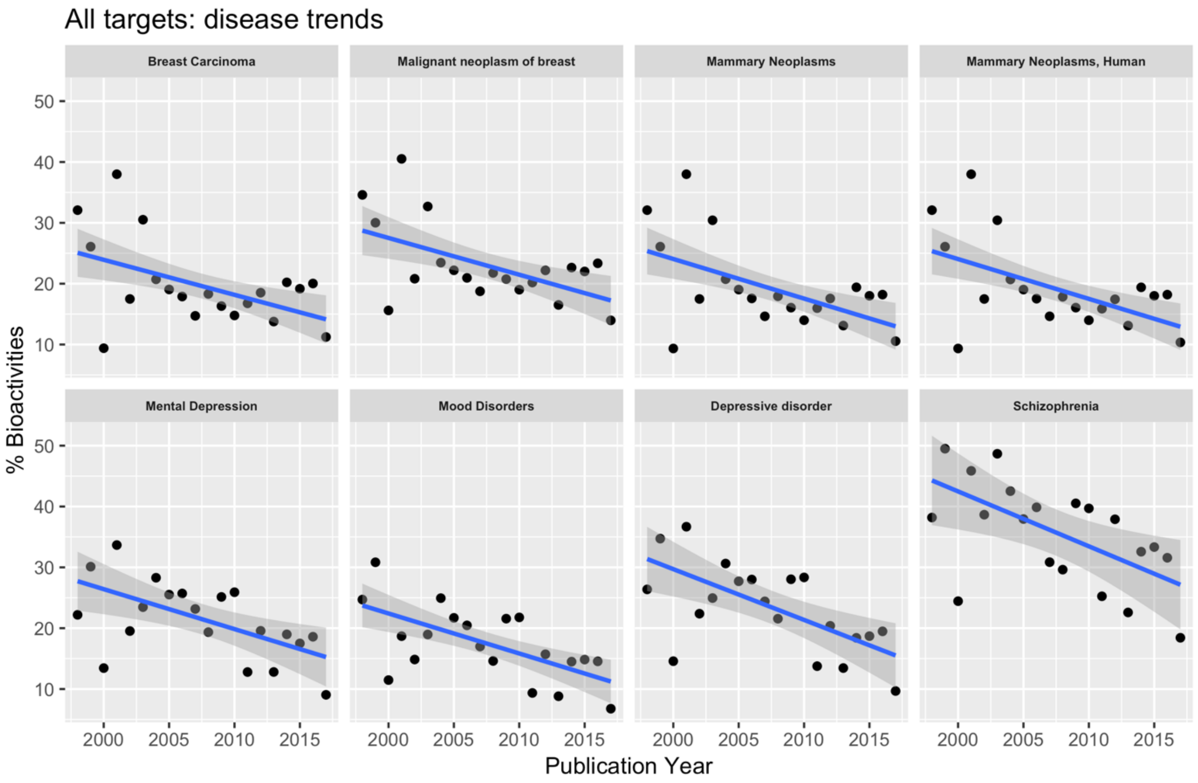

Supplementary Figure S16: *Trend line plots for disease annotations (for the whole data set). Only the eight steepest negative disease trends are shown considering only statistically significant trends (robust regression; $p < 0.05$).*

Supplementary Table S1: *List of most important GO biological process annotations that are annotated to the protein targets under study, including definitions for GO annotations and respective GO IDs (retrieved from https://www.ebi.ac.uk/QuickGO/ and http://www.informatics.jax.org/vocab/gene_ontology/).*

GO biological process annotation	***GO definition***	***GO ID***
anatomical structure development	*The biological process whose specific outcome is the progression of an anatomical structure from an initial condition to its mature state. This process begins with the formation of the structure and ends with the mature structure, whatever form that may be including its natural destruction. An anatomical structure is any biological entity that occupies space and is distinguished from its surroundings. Anatomical structures can be macroscopic such as a carpel, or microscopic such as an acrosome.*	*GO:0048856*
anatomical structure formation involved in morphogenesis	*The developmental process pertaining to the initial formation of an anatomical structure from unspecified parts. This process begins with the specific processes that contribute to the appearance of the discrete structure and ends when the structural rudiment is recognizable. An anatomical structure is any biological entity that occupies space and is distinguished from its surroundings. Anatomical structures can be macroscopic such as a carpel, or microscopic such as an acrosome.*	*GO:0048646*
biosynthetic process	*The chemical reactions and pathways resulting in the formation of substances; typically the energy-requiring part of metabolism in which simpler substances are transformed into more complex ones.*	*GO:0009058*
catabolic process	*The chemical reactions and pathways resulting in the breakdown of substances, including the breakdown of carbon compounds with the liberation of energy for use by the cell or organism.*	*GO:0009056*
cell adhesion	*The attachment of a cell, either to another cell or to an underlying substrate such as the extracellular matrix, via cell adhesion molecules.*	*GO:0007155*
cell cycle	*The progression of biochemical and morphological phases and events that occur in a cell during successive cell replication or nuclear replication events. Canonically, the cell cycle comprises the replication and segregation of genetic material followed by the division of the cell, but in endocycles or syncytial cells nuclear replication or nuclear division may not be followed by cell division.*	*GO:0007049*

cell death	*Any biological process that results in permanent cessation of all vital functions of a cell. A cell should be considered dead when any one of the following molecular or morphological criteria is met: (1) the cell has lost the integrity of its plasma membrane; (2) the cell, including its nucleus, has undergone complete fragmentation into discrete bodies (frequently referred to as apoptotic bodies). The cell corpse (or its fragments) may be engulfed by an adjacent cell in vivo, but engulfment of whole cells should not be considered a strict criteria to define cell death as, under some circumstances, live engulfed cells can be released from phagosomes (see PMID:18045538).*	*GO:0008219*
cell differentiation	*The process in which relatively unspecialized cells, e.g. embryonic or regenerative cells, acquire specialized structural and/or functional features that characterize the cells, tissues, or organs of the mature organism or some other relatively stable phase of the organism's life history. Differentiation includes the processes involved in commitment of a cell to a specific fate and its subsequent development to the mature state.*	*GO:0030154*
cell division	*The process resulting in division and partitioning of components of a cell to form more cells; may or may not be accompanied by the physical separation of a cell into distinct, individually membrane-bounded daughter cells.*	*GO:0051301*
cell morphogenesis	*The developmental process in which the size or shape of a cell is generated and organized.*	*GO:0000902*
cell motility	*Any process involved in the controlled self-propelled movement of a cell that results in translocation of the cell from one place to another.*	*GO:0048870*
cell (population) proliferation	*The multiplication or reproduction of cells, resulting in the expansion of a cell population.*	*GO:0008283*
cell-cell signaling	*Any process that mediates the transfer of information from one cell to another. This process includes signal transduction in the receiving cell and, where applicable, release of a ligand and any processes that actively facilitate its transport and presentation to the receiving cell. Examples include signaling via soluble ligands, via cell adhesion molecules and via gap junctions.*	*GO:0007267*
cellular nitrogen compound metabolic process	*The chemical reactions and pathways involving various organic and inorganic nitrogenous compounds, as carried out by individual cells.*	*GO:0034641*
cellular protein modification process	*The covalent alteration of one or more amino acids occurring in proteins, peptides and nascent polypeptides (co-translational, post-translational modifications) occurring at the level of an individual cell. Includes the modification of charged tRNAs that are destined to occur in a protein (pre-translation modification).*	*GO:0006464*

circulatory system process	*A organ system process carried out by any of the organs or tissues of the circulatory system. The circulatory system is an organ system that moves extracellular fluids to and from tissue within a multicellular organism.*	*GO:0003013*
cytoskeleton organization	*A process that is carried out at the cellular level which results in the assembly, arrangement of constituent parts, or disassembly of cytoskeletal structures.*	*GO:0007010*
embryo development	*The process whose specific outcome is the progression of an embryo from its formation until the end of its embryonic life stage. The end of the embryonic stage is organism-specific. For example, for mammals, the process would begin with zygote formation and end with birth. For insects, the process would begin at zygote formation and end with larval hatching. For plant zygotic embryos, this would be from zygote formation to the end of seed dormancy. For plant vegetative embryos, this would be from the initial determination of the cell or group of cells to form an embryo until the point when the embryo becomes independent of the parent plant.*	*GO:0009790*
extracellular matrix organization	*A process that is carried out at the cellular level which results in the assembly, arrangement of constituent parts, or disassembly of an extracellular matrix.*	*GO:0030198*
growth	*The increase in size or mass of an entire organism, a part of an organism or a cell.*	*GO:0040007*
homeostatic process	*Any biological process involved in the maintenance of an internal steady state.*	*GO:0042592*
immune system process	*Any process involved in the development or functioning of the immune system, an organismal system for calibrated responses to potential internal or invasive threats.*	*GO:0002376*
metabolic process	*The chemical reactions and pathways, including anabolism and catabolism, by which living organisms transform chemical substances. Metabolic processes typically transform small molecules, but also include macromolecular processes such as DNA repair and replication, and protein synthesis and degradation.*	*GO:0008152*
multicellular organism(al) development	*The biological process whose specific outcome is the progression of a multicellular organism over time from an initial condition (e.g. a zygote or a young adult) to a later condition (e.g. a multicellular animal or an aged adult).*	*GO:0007275*
phosphorylation	*The process of introducing a phosphate group into a molecule, usually with the formation of a phosphoric ester, a phosphoric anhydride or a phosphoric amide.*	*GO:0016310*
positive regulation of apoptotic process	*Any process that activates or increases the frequency, rate or extent of cell death by apoptotic process.*	*GO:0043065*
positive regulation of catalytic activity	*Any process that activates or increases the activity of an enzyme.*	*GO:0043085*

protein complex assembly	*The aggregation, arrangement and bonding together of a set of macromolecules to form a protein-containing complex.*	*GO:0065003*
proteolysis	*The hydrolysis of proteins into smaller polypeptides and/or amino acids by cleavage of their peptide bonds.*	*GO:0006508*
regulation of catalytic activity	*Any process that modulates the activity of an enzyme.*	*GO:0050790*
regulation of protein localization	*Any process that modulates the frequency, rate or extent of any process in which a protein is transported to, or maintained in, a specific location.*	*GO:0032880*
reproduction	*The production of new individuals that contain some portion of genetic material inherited from one or more parent organisms.*	*GO:0000003*
response to stress	*Any process that results in a change in state or activity of a cell or an organism (in terms of movement, secretion, enzyme production, gene expression, etc.) as a result of a disturbance in organismal or cellular homeostasis, usually, but not necessarily, exogenous (e.g. temperature, humidity, ionizing radiation).*	*GO:0006950*
signal transduction	*The cellular process in which a signal is conveyed to trigger a change in the activity or state of a cell. Signal transduction begins with reception of a signal (e.g. a ligand binding to a receptor or receptor activation by a stimulus such as light), or for signal transduction in the absence of ligand, signal-withdrawal or the activity of a constitutively active receptor. Signal transduction ends with regulation of a downstream cellular process, e.g. regulation of transcription or regulation of a metabolic process. Signal transduction covers signaling from receptors located on the surface of the cell and signaling via molecules located within the cell. For signaling between cells, signal transduction is restricted to events at and within the receiving cell.*	*GO:0007165*
transmembrane transport	*The process in which a solute is transported across a lipid bilayer, from one side of a membrane to the other.*	*GO:0055085*
transport	*The directed movement of substances (such as macromolecules, small molecules, ions) or cellular components (such as complexes and organelles) into, out of or within a cell, or between cells, or within a multicellular organism by means of some agent such as a transporter, pore or motor protein.*	*GO:0006810*
vesicle-mediated transport	*A cellular transport process in which transported substances are moved in membrane-bounded vesicles; transported substances are enclosed in the vesicle lumen or located in the vesicle membrane. The process begins with a step that directs a substance to the forming vesicle, and includes vesicle budding and coating. Vesicles are then targeted to, and fuse with, an acceptor membrane.*	*GO:0016192*

Supplementary Table S2: *Full list of statistically significant ($p < 0.05$) target-based disease trends retrieved by the study. The (positive or negative) trend is characterized by the slope of the fit (using robust linear regression) of annotation count to year. Only statistically significant trends ($p < 0.05$) are listed.*

Disease	***Slope***	***p-value***
Schizophrenia	*-0.9021*	*0.0032*
Depressive disorder	*-0.8344*	*0.0008*
Mood Disorders	*-0.6579*	*0.0090*
Mental Depression	*-0.6564*	*0.0057*
Mammary Neoplasms, Human	*-0.6538*	*0.0055*
Mammary Neoplasms	*-0.6511*	*0.0057*
Malignant neoplasm of breast	*-0.6042*	*0.0069*
Breast Carcinoma	*-0.5755*	*0.0160*
Unipolar Depression	*-0.5720*	*0.0083*
Major Depressive Disorder	*-0.5414*	*0.0040*
Colorectal Cancer	*-0.5264*	*0.0015*
Anaplastic carcinoma	*-0.4734*	*0.0000*
Carcinoma	*-0.4734*	*0.0000*
Carcinoma, Spindle-Cell	*-0.4734*	*0.0000*
Carcinomatosis	*-0.4734*	*0.0000*
Undifferentiated carcinoma	*-0.4734*	*0.0000*
Hypertensive disease	*-0.4670*	*0.0001*
Myocardial Infarction	*-0.4375*	*0.0000*
Adverse reaction to drug	*-0.4301*	*0.0003*
Drug toxicity	*-0.4301*	*0.0003*
Liver Cirrhosis, Experimental	*-0.4190*	*0.0048*
Alcoholic Intoxication, Chronic	*-0.3945*	*0.0420*
Reperfusion Injury	*-0.3748*	*0.0002*
Alcohol abuse	*-0.3686*	*0.0191*
Absence Seizures	*-0.3614*	*0.0302*
Atonic Absence Seizures	*-0.3614*	*0.0302*
Complex partial seizures	*-0.3614*	*0.0302*
Convulsions	*-0.3614*	*0.0302*

Convulsive Seizures	*-0.3614*	*0.0302*
Epileptic Seizures	*-0.3614*	*0.0302*
Epileptic drop attack	*-0.3614*	*0.0302*
Generalized Absence Seizures	*-0.3614*	*0.0302*
Generalized seizures	*-0.3614*	*0.0302*
Gustatory seizure	*-0.3614*	*0.0302*
Jacksonian Seizure	*-0.3614*	*0.0302*
Myoclonic Seizures	*-0.3614*	*0.0302*
Non-epileptic convulsion	*-0.3614*	*0.0302*
Nonepileptic Seizures	*-0.3614*	*0.0302*
Olfactory seizure	*-0.3614*	*0.0302*
Seizures	*-0.3614*	*0.0302*
Seizures, Auditory	*-0.3614*	*0.0302*
Seizures, Clonic	*-0.3614*	*0.0302*
Seizures, Focal	*-0.3614*	*0.0302*
Seizures, Sensory	*-0.3614*	*0.0302*
Seizures, Somatosensory	*-0.3614*	*0.0302*
Single Seizure	*-0.3614*	*0.0302*
Tonic - clonic seizures	*-0.3614*	*0.0302*
Tonic Seizures	*-0.3614*	*0.0302*
Vertiginous seizure	*-0.3614*	*0.0302*
Visual seizure	*-0.3614*	*0.0302*
Liver carcinoma	*-0.3583*	*0.0006*
Obesity	*-0.3519*	*0.0073*
Rheumatoid Arthritis	*-0.3408*	*0.0002*
Neoplasm Metastasis	*-0.3332*	*0.0032*
Cocaine Dependence	*-0.3328*	*0.0173*
Allodynia	*-0.3206*	*0.0008*
Hyperalgesia	*-0.3206*	*0.0008*
Hyperalgesia, Primary	*-0.3206*	*0.0008*
Hyperalgesia, Secondary	*-0.3206*	*0.0008*
Hyperalgesia, Thermal	*-0.3206*	*0.0008*
Mechanical Allodynia	*-0.3206*	*0.0008*
Tactile Allodynia	*-0.3206*	*0.0008*
Cocaine Abuse	*-0.3067*	*0.0135*

Alloxan Diabetes	*-0.2971*	*0.0000*
Diabetes Mellitus, Experimental	*-0.2971*	*0.0000*
Streptozotocin Diabetes	*-0.2971*	*0.0000*
Marijuana Abuse	*-0.2839*	*0.0144*
Colorectal Carcinoma	*-0.2829*	*0.0119*
Colorectal Neoplasms	*-0.2829*	*0.0119*
Esophageal Neoplasms	*-0.2736*	*0.0018*
Malignant neoplasm of esophagus	*-0.2736*	*0.0018*
Cocaine-Related Disorders	*-0.2632*	*0.0211*
Peripheral Neuropathy	*-0.2622*	*0.0023*
Animal Mammary Neoplasms	*-0.2531*	*0.0028*
Mammary Carcinoma, Animal	*-0.2531*	*0.0028*
Familial M,àö¬©ni,àö¬Ære disease	*-0.2443*	*0.0001*
Bradycardia	*-0.2381*	*0.0008*
Coronary Arteriosclerosis	*-0.2362*	*0.0000*
Coronary Artery Disease	*-0.2362*	*0.0000*
Mammary Neoplasms, Experimental	*-0.2343*	*0.0027*
Psychoses, Substance-Induced	*-0.2234*	*0.0017*
Ischemia	*-0.2232*	*0.0000*
Neoplasm Invasiveness	*-0.2230*	*0.0068*
Endometrioma	*-0.2224*	*0.0213*
Endometriosis	*-0.2224*	*0.0213*
Psychoses, Drug	*-0.2210*	*0.0021*
Chemical and Drug Induced Liver Injury	*-0.2200*	*0.0015*
Chemically-Induced Liver Toxicity	*-0.2200*	*0.0015*
Drug-Induced Acute Liver Injury	*-0.2200*	*0.0015*
Drug-Induced Liver Disease	*-0.2200*	*0.0015*
Hepatitis, Drug-Induced	*-0.2200*	*0.0015*
Hepatitis, Toxic	*-0.2200*	*0.0015*
Arthritis, Adjuvant-Induced	*-0.2126*	*0.0010*
Arthritis, Collagen-Induced	*-0.2126*	*0.0010*
Arthritis, Experimental	*-0.2126*	*0.0010*
Astrocytoma	*-0.2066*	*0.0001*
nervous system disorder	*-0.2009*	*0.0003*
Colonic Neoplasms	*-0.1987*	*0.0215*

Aura	*-0.1971*	*0.0023*
Awakening Epilepsy	*-0.1971*	*0.0023*
Epilepsy	*-0.1971*	*0.0023*
Epilepsy, Cryptogenic	*-0.1971*	*0.0023*
Cerebral Hemorrhage	*-0.1886*	*0.0044*
Anhedonia	*-0.1883*	*0.0167*
Atherogenesis	*-0.1877*	*0.0287*
Atherosclerosis	*-0.1877*	*0.0287*
Depression, Bipolar	*-0.1857*	*0.0001*
Cerebral Astrocytoma	*-0.1761*	*0.0005*
Childhood Cerebral Astrocytoma	*-0.1761*	*0.0005*
Diffuse Astrocytoma	*-0.1761*	*0.0005*
Fibrillary Astrocytoma	*-0.1761*	*0.0005*
Gemistocytic astrocytoma	*-0.1761*	*0.0005*
Grade I Astrocytoma	*-0.1761*	*0.0005*
Intracranial Astrocytoma	*-0.1761*	*0.0005*
Juvenile Pilocytic Astrocytoma	*-0.1761*	*0.0005*
Mixed oligoastrocytoma	*-0.1761*	*0.0005*
Protoplasmic astrocytoma	*-0.1761*	*0.0005*
Subependymal Giant Cell Astrocytoma	*-0.1761*	*0.0005*
Non-alcoholic Fatty Liver Disease	*-0.1751*	*0.0001*
Nonalcoholic Steatohepatitis	*-0.1751*	*0.0001*
Pilocytic Astrocytoma	*-0.1743*	*0.0004*
Anaplastic astrocytoma	*-0.1731*	*0.0009*
Congestive heart failure	*-0.1632*	*0.0310*
Heart Decompensation	*-0.1632*	*0.0310*
Heart Failure, Right-Sided	*-0.1632*	*0.0310*
Heart failure	*-0.1632*	*0.0310*
Left-Sided Heart Failure	*-0.1632*	*0.0310*
Myocardial Failure	*-0.1632*	*0.0310*
Neoplasm Recurrence, Local	*-0.1619*	*0.0017*
Hypotension, Orthostatic	*-0.1614*	*0.0025*
Fatty Liver	*-0.1599*	*0.0003*
Steatohepatitis	*-0.1599*	*0.0003*
Brain Ischemia	*-0.1583*	*0.0037*

Cerebral Ischemia	*-0.1583*	*0.0037*
Poisoning	*-0.1576*	*0.0006*
Disease Exacerbation	*-0.1574*	*0.0151*
Drug Allergy	*-0.1492*	*0.0281*
Hyperactive behavior	*-0.1482*	*0.0393*
Hyperkinesia, Generalized	*-0.1482*	*0.0393*
Endometrial Neoplasms	*-0.1432*	*0.0365*
Action Tremor	*-0.1413*	*0.0030*
Coarse Tremor	*-0.1413*	*0.0030*
Continuous Tremor	*-0.1413*	*0.0030*
Darkness Tremor	*-0.1413*	*0.0030*
Fine Tremor	*-0.1413*	*0.0030*
Intermittent Tremor	*-0.1413*	*0.0030*
Involuntary Quiver	*-0.1413*	*0.0030*
Massive Tremor	*-0.1413*	*0.0030*
Nerve Tremors	*-0.1413*	*0.0030*
Passive Tremor	*-0.1413*	*0.0030*
Persistent Tremor	*-0.1413*	*0.0030*
Pill Rolling Tremor	*-0.1413*	*0.0030*
Resting Tremor	*-0.1413*	*0.0030*
Saturnine Tremor	*-0.1413*	*0.0030*
Senile Tremor	*-0.1413*	*0.0030*
Static Tremor	*-0.1413*	*0.0030*
Tremor	*-0.1413*	*0.0030*
Tremor, Limb	*-0.1413*	*0.0030*
Tremor, Muscle	*-0.1413*	*0.0030*
Tremor, Neonatal	*-0.1413*	*0.0030*
Tremor, Perioral	*-0.1413*	*0.0030*
Tremor, Semirhythmic	*-0.1413*	*0.0030*
Nerve Degeneration	*-0.1407*	*0.0261*
Female infertility	*-0.1388*	*0.0018*
Female sterility	*-0.1388*	*0.0018*
Sterility, Postpartum	*-0.1388*	*0.0018*
Subfertility, Female	*-0.1388*	*0.0018*
Cannabis Dependence	*-0.1355*	*0.0196*

Chronic Airflow Obstruction	*-0.1345*	*0.0016*
Chronic Obstructive Airway Disease	*-0.1345*	*0.0016*
Neutropenia	*-0.1321*	*0.0077*
Depression, Postpartum	*-0.1299*	*0.0017*
Nausea	*-0.1281*	*0.0015*
Alcoholic Intoxication	*-0.1276*	*0.0339*
Major depression, single episode	*-0.1240*	*0.0258*
Congenital Heart Defects	*-0.1211*	*0.0045*
Manic Disorder	*-0.1195*	*0.0008*
Encephalopathy, Toxic	*-0.1152*	*0.0218*
Neurotoxicity Syndromes	*-0.1152*	*0.0218*
Toxic Encephalitis	*-0.1152*	*0.0218*
Manic	*-0.1149*	*0.0019*
Fever	*-0.1053*	*0.0243*
Kidney Diseases	*-0.1038*	*0.0166*
Pyemia	*-0.0984*	*0.0110*
Sepsis	*-0.0984*	*0.0110*
Septicemia	*-0.0984*	*0.0110*
Severe Sepsis	*-0.0984*	*0.0110*
Acute Cerebrovascular Accidents	*-0.0980*	*0.0143*
Cerebrovascular accident	*-0.0980*	*0.0143*
Schizoaffective Disorder	*-0.0963*	*0.0177*
Male infertility	*-0.0949*	*0.0128*
Male sterility	*-0.0949*	*0.0128*
Subfertility, Male	*-0.0949*	*0.0128*
Embolic Infarction, Middle Cerebral Artery	*-0.0920*	*0.0029*
Infarction, Middle Cerebral Artery	*-0.0920*	*0.0029*
Left Middle Cerebral Artery Infarction	*-0.0920*	*0.0029*
Middle Cerebral Artery Embolus	*-0.0920*	*0.0029*
Middle Cerebral Artery Occlusion	*-0.0920*	*0.0029*
Middle Cerebral Artery Syndrome	*-0.0920*	*0.0029*
Middle Cerebral Artery Thrombosis	*-0.0920*	*0.0029*
Right Middle Cerebral Artery Infarction	*-0.0920*	*0.0029*
Thrombotic Infarction, Middle Cerebral Artery	*-0.0920*	*0.0029*
Brain Injuries	*-0.0892*	*0.0245*

Brain Injuries, Focal	*-0.0892*	*0.0245*
Brain Lacerations	*-0.0892*	*0.0245*
Injuries, Acute Brain	*-0.0892*	*0.0245*
Cannabis Abuse	*-0.0867*	*0.0129*
Cannabis-Related Disorder	*-0.0867*	*0.0129*
Hashish Abuse	*-0.0867*	*0.0129*
Adrenocortical carcinoma	*-0.0755*	*0.0433*
Osteosarcoma	*0.1052*	*0.0125*
Breast adenocarcinoma	*0.1159*	*0.0131*
Neoplasm of uncertain or unknown behavior of breast	*0.1159*	*0.0131*
Squamous cell carcinoma of lung	*0.1163*	*0.0113*
Glioma	*0.1246*	*0.0325*
Squamous cell carcinoma	*0.1275*	*0.0334*
Conventional (Clear Cell) Renal Cell Carcinoma	*0.1340*	*0.0493*
Chromophobe Renal Cell Carcinoma	*0.1368*	*0.0467*
Collecting Duct Carcinoma of the Kidney	*0.1368*	*0.0467*
Papillary Renal Cell Carcinoma	*0.1368*	*0.0467*
Renal Cell Carcinoma	*0.1368*	*0.0467*
Sarcomatoid Renal Cell Carcinoma	*0.1368*	*0.0467*
Experimental Hepatoma	*0.1429*	*0.0099*
Hepatoma, Morris	*0.1429*	*0.0099*
Hepatoma, Novikoff	*0.1429*	*0.0099*
Liver Neoplasms, Experimental	*0.1429*	*0.0099*
Malignant neoplasm of ovary	*0.1656*	*0.0022*
ovarian neoplasm	*0.1817*	*0.0005*
melanoma	*0.2137*	*0.0017*
Cutaneous Melanoma	*0.2343*	*0.0000*
Acute Myeloid Leukemia, M1	*0.2406*	*0.0000*
Epileptic encephalopathy	*0.2412*	*0.0000*
Acute Myeloid Leukemia (AML-M2)	*0.2618*	*0.0000*
Adenocarcinoma of lung (disorder)	*0.2813*	*0.0004*
Adenocarcinoma of large intestine	*0.2853*	*0.0026*
Leukemia, Myelocytic, Acute	*0.2936*	*0.0000*
Intellectual Disability	*0.5981*	*0.0000*
